# Grass Carp Laboratory of Genetics and Physiology 2 Serves As a Negative Regulator in Retinoic Acid-Inducible Gene I- and Melanoma Differentiation-Associated Gene 5-Mediated Antiviral Signaling in Resting State and Early Stage of Grass Carp Reovirus Infection

**DOI:** 10.3389/fimmu.2017.00352

**Published:** 2017-03-27

**Authors:** Youliang Rao, Quanyuan Wan, Chunrong Yang, Jianguo Su

**Affiliations:** ^1^College of Fisheries, Huazhong Agricultural University, Wuhan, China; ^2^College of Veterinary Medicine, Huazhong Agricultural University, Wuhan, China

**Keywords:** laboratory of genetics and physiology 2, innate immunity, grass carp (*Ctenopharyngodon idella*), grass carp reovirus, RLRs, interferon regulatory factor 3, IRF7

## Abstract

Laboratory of genetics and physiology 2 (LGP2) is a key component of RIG-I-like receptors (RLRs). However, the lack of the caspase recruitment domains (CARDs) results in its controversial functional performance as a negative or positive regulator in antiviral responses. Especially, no sufficient evidence uncovers the functional mechanisms of LGP2 in RLR signaling pathways in teleost. Here, negative regulation mechanism of LGP2 in certain situations in retinoic acid-inducible gene I (RIG-I) and melanoma differentiation-associated gene 5 (MDA5)-mediated antiviral responses was identified in *Ctenopharyngodon idella* kidney cells. LGP2 overexpression inhibits synthesis and phosphorylation of interferon regulatory factor 3/7 (IRF3/7), and mRNA levels and promoter activities of IFNs and NF-κBs in resting state and early phase of grass carp reovirus (GCRV) infection. Knockdown of LGP2 obtains opposite effects. Luciferase report assay indicates that LGP2 works at the upstream of RIG-I and MDA5. LGP2 binds to RIG-I and MDA5 with diverse domain preference and which is independent of GCRV infection. Furthermore, LGP2 restrains K63-linked ubiquitination of RIG-I and MDA5 in various degrees. These differences result in disparate repressive mechanisms of LGP2 to RIG-I- and MDA5-mediated signal activations of IFN-β promoter stimulator 1 and mediator of IRF3 activation. Interestingly, LGP2 also inhibits K48-linked RIG-I and MDA5 ubiquitination to suppress proteins degradation, which guarantees the basal protein levels for subsequently rapid signal activation. All these results reveal a mechanism that LGP2 functions as a suppressor in RLR signaling pathways to maintain cellular homeostasis in resting state and early phase during GCRV infection.

## Highlights

1. LGP2 interacts with RIG-I and MDA5 independent of GCRV infection.
2. LGP2 suppresses both K63- and K48-linked ubiquitination of RIG-I and MDA5.
3. LGP2 restrains activation of IRF3/7 *via* repressing their Ser and Thr phosphorylation.
4. LGP2 inhibits production and promoter activities of IFNs and NF-κBs in resting state and early stage of GCRV infection.
5. Knockdown of LGP2 enhances the immune responses of IFNs, NF-κBs, and IRF3/7.

## Introduction

The host possesses intrinsic antiviral immune system that binds viral components and inhibits viral replication (1). Pattern recognition receptors (PRRs) directly sense the presence of pathogen components, so called pathogen-associated molecular patterns (PAMPs) (2, 3). RIG-I like receptors (RLRs) are a family of cytoplasmic PRRs that sense viral PAMPs in cytosol (1, 3, 4). Three members have been identified in this family: retinoic acid-inducible gene I (RIG-I), melanoma differentiation-associated gene 5 (MDA5), and laboratory of genetics and physiology 2 (LGP2), and all of them belong to DExD/H box RNA helicases family (5). RIG-I and MDA5 have three domains: two tandem N-terminal caspase recruitment domains (CARDs), a DExD/H-box helicase, and a C-terminal repressor domain (RD). LGP2 has DExD/H-box helicase and RD domains, but lacks the CARD (6). To date, RIG-I and MDA5 have been well characterized: RIG-I mainly recognizes RNAs with 5’ PPP or short dsRNA (~20 bp), while RNA web can induce the activation of MDA5 (1, 7). Both RIG-I and MDA5 can sense a wide variety of RNA or DNA viruses (1, 7). Upon viral recognition, RIG-I and MDA5 activate nuclear factor κB (NF-κB) and interferon regulatory factor 3 (IRF3) through the adaptor proteins IFN-β promoter stimulator 1 [(IPS-1), also known as MAVS, VISA, or Cardif] and mediator of IRF3 activation [MITA, also named as STING] (8, 9). IPS-1 is a CARD domain-containing protein that drives the expression of type I interferons (IFN-Is) and inflammatory cytokines (1). MITA functions downstream of RIG-I and IPS-1, which is necessary for efficient induction of IFN-Is and IFN-stimulated genes (ISGs) (8, 10).

As for the third member of RLRs, LGP2 lacks the CARDs, which implies the different functions from RIG-I and MDA5. Up to now, the role of LGP2 in antiviral signaling is controversial. Accumulating data report the antithetical roles of LGP2 as a negative or positive regulator in antiviral responses (11–13). A study indicates that LGP2 RD is necessary and sufficient for inhibition of RIG-I, but not MDA5, by interacting *in trans* with RIG-I to ablate self-association and signaling (14). Moreover, LGP2 can inhibit antiviral signaling by competing with the kinase IKKi for a common interaction site on IPS-1 (15). However, other groups support the positive role of LGP2 in antiviral responses (16–19). Direct evidence suggests that LGP2 assists MDA5–RNA interaction and regulates MDA5 filament assembly to enhance MDA5-mediated antiviral signaling (16). LGP2 can synergize with MDA5 to potentiate IFN-β transcription *in vivo* during encephalomyocarditis virus infection or polyinosinic–polycytidylic potassium salt [poly(I:C)] transfection *via* ATP-enhanced RNA recognition (17). LGP2 also facilitates viral RNA recognition by both RIG-I and MDA5 through its ATPase domain (18). In the Chinese tree shrew, LGP2 synergizes with MDA5 to sense Sendai virus infection for IFN-I induction along with the loss of RIG-I (19).

Fish harbor more complicated innate immune systems than those in mammals (20). Nearly all the counterparts of vertebrate PRRs and their downstream signaling components have been identified in teleost (20, 21). RLRs are evolutionarily conserved from fish to mammals (21). Generally, teleost RLRs also consist of three members: RIG-I, MDA5, and LGP2, although RIG-I is absent in some fish species (20). So far, RLRs have been identified in many teleosts such as zebrafish (*Danio rerio*) (21–23), grass carp (*Ctenopharyngodon idella*) (24–26), rainbow trout (*Oncorhynchus mykiss*) (12), Atlantic salmon (*Salmo salar*), and Japanese flounder (*Paralichthys olivaceus*) (27). The roles of RLRs in mediating downstream signal pathways have been preliminarily studied in some fish species (28–31). Nevertheless, the antithetical functions of fish LGP2 as a positive or negative regulator in antiviral responses are still controversial in fish (12, 32–34).

Grass carp is an important freshwater economic fish in China. However, hemorrhagic disease caused by grass carp reovirus (GCRV), a dsRNA virus, seriously affects the grass carp cultivation industry (20). To uncover the definite role of grass carp LGP2 in antiviral immune responses, the regulation mechanisms of LGP2 in RLR signaling pathways in response to GCRV infection were investigated in *Ctenopharyngodon idella* kidney (CIK) cells. Our results demonstrated that grass carp LGP2 function as a negative regulator in RIG-I and MDA5-mediated antiviral immune responses under resting state and early phase of GCRV infection. The findings provide a molecular mechanism on LGP2 in maintaining cellular homeostasis and preventing the host from the uncontrolled innate immune responses.

## Materials and Methods

### Cells and Virus Infection

*Ctenopharyngodon idella* kidney cells, obtained from China Center for Type Culture Collection, were cultured according to previous description (35). Fathead minnow (FHM) cell line (36) was kindly provided by Dr. Junfa Yuan, which was maintained in M199 (Gibco) supplemented with 10% FBS (Gibco), 100 U/ml of penicillin (Sigma), and 100 U/ml of streptomycin (Sigma). Both cells were incubated at 28°C with 5% CO_2_ humidified atmosphere.

For virus infection, CIK or FHM cells were plated for 24 h in advance and then infected with GCRV 097 stain at a multiplicity of infection of 1. After 2 h, the virus inoculum was removed, the cells were washed with PBS, and further incubated with new medium (DMEM for CIK, M199 for FHM, and no FBS). The control group was treated with PBS.

### Plasmid Constructions and Transfections

pCMV-CMV-GFP was employed as original plasmid (28) for constructing the following expression plasmids: LGP2-Flag, RIG-I-Flag, MDA5-Flag, RIG-I-HA, RIG-I-CARD-HA, RIG-I-Helicase-HA, RIG-I-RD-HA, MDA5-HA, MDA5-CARD-HA, MDA5-Helicase-HA, MDA5-RD-HA, RIG-I-CARD-Flag, and MDA5-CARD-Flag. The ORFs or the domains of the relevant genes were amplified from grass carp spleen tissue cDNA and then inserted behind the first CMV promoter. The Flag or HA tag was introduced by the reverse primer. The primers were listed in Table S1 in Supplementary Material. To construct the luciferase reporter vectors of grass carp IPS-1, MITA, IRF3, IRF7, IFN1, IFN2, IFN3, IFN4, IFNγ1, IFNγ2, NF-κB1, and NF-κB2, the 5′-flanking fragments of these genes were obtained from the grass carp genome (37). The core promoter regions were predicted by WWW Promoter Scan,^1^ GPMiner,^2^ and Promoter 2.0 Prediction Server.^3^ To verify the promoter activities, the predicted promoters of the related genes were introduced to the pCMV-GFP vector by replacing the CMV promoter (38). The primers were shown in Table S1 in Supplementary Material. Then, these vectors were transfected into CIK cells by FuGENE 6 Transfection Reagent (Promega), respectively. The promoter activity was reflected by promoting green fluorescent protein expression, observed under a fluorescence microscope (Nikon). The promoter activities of RIG-I, MDA5, and MITA were verified in the previous studies (39–41). For dual-luciferase reporter assay, the valid promoters were cloned into pGL3-basic luciferase reporter vector (Promega), respectively. For transient transfection, CIK or FHM cells were plated in 24-well plates, 6-well plates, or 10 cm^2^ dishes with 70–90% confluency. Approximately 24 h later, transfection was performed with FuGENE 6 Transfection Reagent (Promega) according to the manufacturer’s instructions. LGP2 stable transfected cell line was obtained by G418 selection as previously reported (38). It is worth noting that the vector (pCMV-CMV-EGFP) used for protein overexpression in the present study contains two CMV promoters, which promote the expressions of target protein and EGFP, respectively, and the later is used to monitor the transfection efficiency. Hence, we employed empty vector-transfected cells rather than normal cells as control in the present study, which can make a better demonstration of LGP2 functions in RLR signaling pathways, not EGFP or other components in the vector skeleton. To assess the influence of empty vector on dual luciferase reporter assay, transcription level, and protein expression, we compared the promoter activities, mRNA expressions, and protein levels between empty vector-transfected cells and normal cells, and the results demonstrated that empty vector has no significant influence on the promoter activity, mRNA level, and protein synthesis (Figures S1 and S2 in Supplementary Material; Figure 3E).

### Dual Luciferase Reporter Assays

Fathead minnow cells were seeded in 24-well plates and cotransfected with the indicated luciferase reporter plasmid and overexpression plasmid. pRL-TK vector (Promega) was used as an internal control to normalize the expression level of the transfected plasmid. At 16 h post-transfection, the cells were infected with GCRV or treated with PBS for 12 or 24 h, then washed with PBS, and lysed by Passive Lysis Buffer (Promega). Dual-luciferase reporter assay was conducted in 96-well luminometer plates with Dual-Luciferase Reporter Assay System according to the manufacturer’s instructions (Promega). Luciferase activity was measured by Multiscan Spectrum (PerkinElmer). Data represent relative firefly luciferase activity normalized to Renilla luciferase activity. The results were obtained from four independent experiments, and each was performed in triplicate.

### Immunoprecipitation (IP) and Western Blotting (WB) Analyses

For transient transfection and Co-IP experiments, FHM cells in 10 cm^2^ dishes were co-transfected with the indicated plasmids for 24 h, then infected with GCRV for 12 or 24 h according to test requirements. The cells were lysed in western and IP lysis buffer (20 mM Tris (pH 7.4), 150 mM NaCl, 1% Triton X-100, 1mM EDTA, 1mM Na3VO4, 0.5 µg/ml leupeptin, 2.5 mM sodium pyrophosphate) (Beytotime) added with 1 mM PMSF for 30 min on ice, and then centrifuged at 12,000 rpm for 30 min at 4°C. For each IP, 1 mg of cell lysate was incubated with 1 µg of the indicated antibody (Ab) overnight at 4°C, adding 35 µl of protein A + G-agarose (Beyotime) for 4 h. The sepharose beads were washed three times with 1 ml lysis buffer, then eluted with 20 µl 2 × SDS loading buffer by boiling for 10 min at 95°C. The precipitates were detected by IP with indicated Ab.

For WB, protein extracts were separated by 8% SDS-PAGE gels and transferred onto NC membranes (Millipore). The membranes were blocked in fresh 3% non-fat dry milk dissolved in TBST buffer for 2 h at room temperature, then incubated with the following primary Ab for 2 h at room temperature: anti-Flag (monoclonal, 1:1,000) (Abcam), anti-HA (monoclonal, 1:1,000) (Abcam), anti-β-Tubulin (monoclonal, 1:5,000) (Abcam), respectively. Rabbit polyclonal antiserum of IRF3 was kindly provided by Prof. Yibing Zhang. Anti-IRF7 rabbit antiserum was prepared in our laboratory. Purified rabbit polyclonal anti-phosphoserine (anti-pSer), anti-phosphothreonine (anti-pThr), and anti-phosphotyrosine (anti-pTyr) Ab were purchased from IMMUNECHEM (Canada). Calf intestinal alkaline phosphatase (CIP) used for dephosphorylation was purchased from BioLabs. The results were obtained from three independent experiments.

*In vivo* ubiquitination assay was performed in FHM cells. The transiently transfected cells were infected with GCRV at indicated time points and treated with 25 µM MG132 (Selleckchem) for 6 h before harvest, then lysed with NP-40 lysis buffer [50 mM Tris (pH 7.4), 150 mM NaCl, 1% NP-40, 1mM EDTA, 1mM Na_3_VO_4_, 0.5 µg/ml leupeptin, 2.5 mM sodium pyrophosphate] (Beytotime) added with 1 mM PMSF and 1% SDS for 30 min on ice. Before centrifugation, the samples were diluted with lysis buffer to ensure the final concentration of SDS with 0.1%. IP and immunoblotting (IB) examinations were conducted as above descriptions.

### siRNA-Mediated Knockdown

Transient knockdown of endogenous LGP2 in CIK cells were achieved by transfection of siRNA targeting on LGP2 mRNA. Three siRNA sequences (s1: AAAGUGCUGGUCUACCAGG, s2: CCUGGUAGACCAGCACUUU, s3: AUCUUCAAAGGUCUUCUCC) targeting different regions of LGP2 gene were synthesized by RiboBio. The silencing efficiencies of the three LGP2 siRNA candidates were evaluated by qRT-PCR and WB, comparing with those in the negative control siRNA provided by the supplier. Our preliminary experiment indicated that s3 possesses the best silencing efficiency at a final concentration of 100 nM in mRNA level. For WB, LGP2-Flag overexpression CIK cell line was plated in 6-well plates and transfected with s3 using FuGENE 6. The cells were lysed for WB at 48 h post-transfection.

### qRT-PCR

Total RNAs were isolated using RNAiso Plus (TaKaRa) according to manufacturer’s instructions and incubated with RNase-free DNase I to eliminate contaminated genomic DNA. Reverse transcription was performed using random hexamer primers and M-MLV Reverse Transcriptase (Promega). Roche LightCycler^®^ 480 system was used to quantify the mRNA expressions of related genes. EF1α was employed as an internal control gene for cDNA normalization (42). The qRT-PCR amplification was carried out in a total volume of 15 µl, containing 7.5 µl of BioEasy Master Mix (SYBR Green) (Hangzhou Bioer Technology Co., Ltd.), 5.1 µl of nuclease-free water, 2 µl of diluted cDNA (200 ng), and 0.2 µl of each gene specific primer (10 µM). The relative mRNA abundances were calculated using the 2^−ΔΔCT^ method and normalized to EF1α. All data were subjected to one-way analysis of variance (one-way ANOVA), followed by unpaired, two-tailed *t*-test (35). The results were obtained from four independent experiments, and each was performed in triplicate.

## Results

### Identification of LGP2 As a Negative Mediator in IFNs and NF-κBs Induction

**Figure 1:**
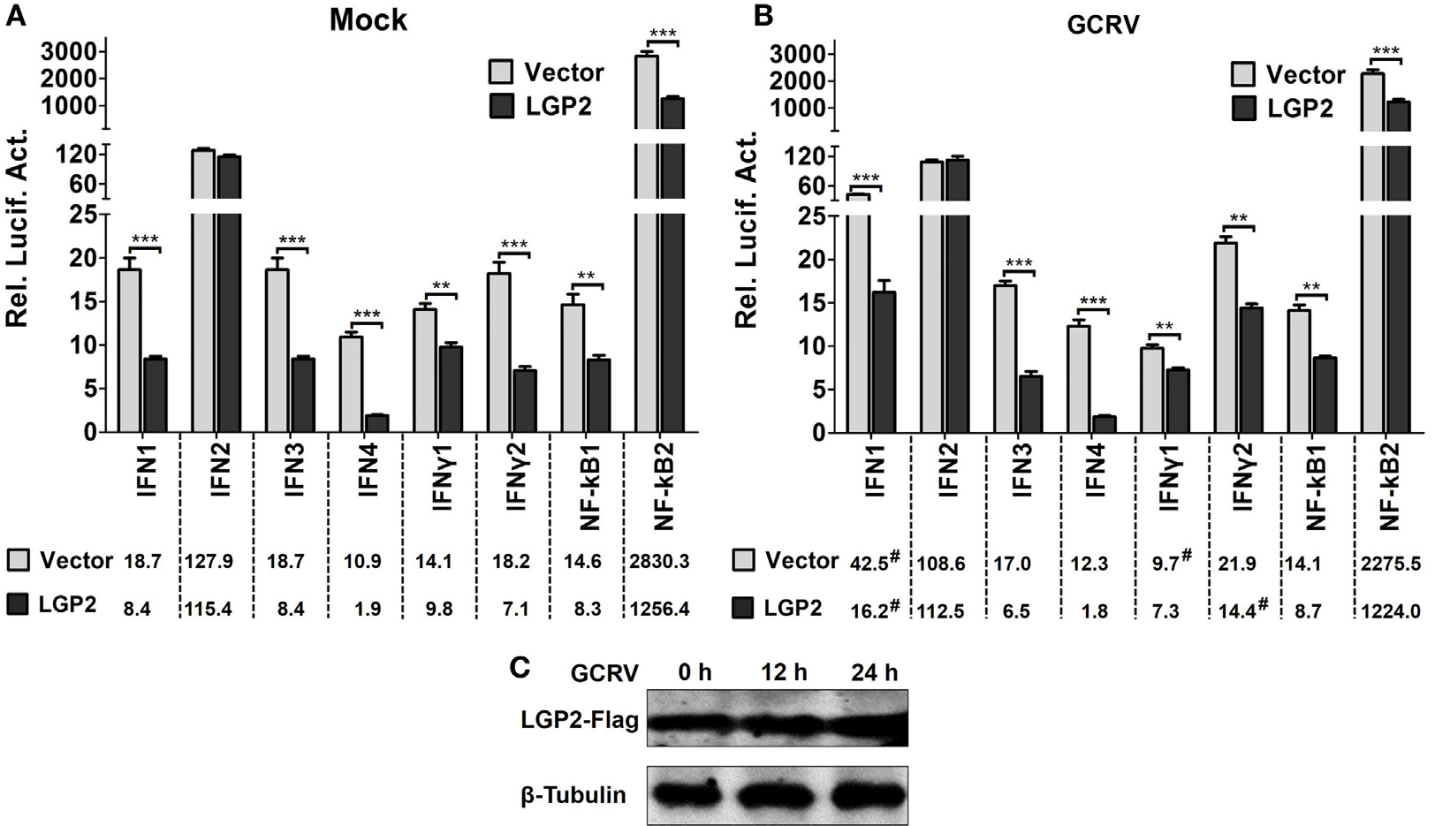
**Identification of laboratory of genetics and physiology 2 (LGP2) as an inhibitor in IFNs and NF-κBs activation**. **(A,B)** LGP2 overexpression suppresses the promoter activities of IFNs and NF-κBs. Fathead minnow (FHM) cells were cotransfected with 300 ng of LGP2-Flag overexpression plasmid, 30 ng of pRL-TK, and 300 ng of IFN1pro-luc, IFN2pro-luc, IFN3pro-luc, IFN4pro-luc, IFNγ1pro-luc, IFNγ2pro-luc, NF-κB1pro-luc, NF-κB2pro-luc in 24-well plates. Control was transfected with 300 ng of empty vector (pCMV-CMV-GFP), same amount of the corresponding report vectors, and pRL-TK. At 16 h post-transfection, the cells were infected with grass carp reovirus (GCRV) or uninfected. Dual-luciferase report assays were conducted at 12 h (IFN2, IFN4, IFNγ2) or 24 h (IFN1, IFN3, IFNγ1, NF-κB1, NF-κB2) after GCRV infection. Time-matched mocks were treated with PBS. Error bars indicate SD (*n* = 4). Asterisks indicate significant differences from control (**0.001 < *P* < 0.01; ***P < 0.001). Digitals under histograms show the average values. Symbol “#” indicates significant difference between mock and GCRV-infected conditions. **(C)** Examination of exogenous LGP2 in FHM cells upon GCRV infection. FHM cells were transfected with 1 µg of LGP2-Flag overexpression plasmid in 6-well plates. GCRV infection was preformed at 12 and 24 h post-transfection. The cell lysates were prepared for WB using anti-Flag and anti-β-Tubulin Abs.

IFNs and NF-κBs are effector molecules mainly involved in virus-triggered innate immune responses. To identify the roles of LGP2 in GCRV-mediated IFNs and NF-κBs induction, we examined the promoter activities of all the members of grass carp IFNs (type I IFNs: IFN1, IFN2, IFN3, IFN4; type II IFNs: IFNγ1, IFNγ2) (43) and NF-κBs (NF-κB1, NF-κB2) upon LGP2 overexpression. In all these effector molecules, IFN2, IFN4, and IFNγ2 are early induced, so their promoter activities were examined at 12 h post-GCRV inoculation, and the others were investigated at 24 h. As shown in Figures 1A,B, except for IFN2, the promoter activities of all the examined genes were significantly suppressed in LGP2 overexpression cells under mock and GCRV-infected conditions. The promoter activities of IFN1, IFNγ1, and IFNγ2 were remarkably changed upon GCRV infection in empty vector or LGP2 transfected cells. GCRV infection had no influence on the protein level of exogenous LGP2 in FHM cells (Figure 1C). These results indicate that LGP2 plays a negative role in both IFNs and NF-κBs pathways.

### LGP2 Inhibits the Expressions of IFNs and NF-κBs at the Early Phase Post-GCRV Infection

**Figure 2:**
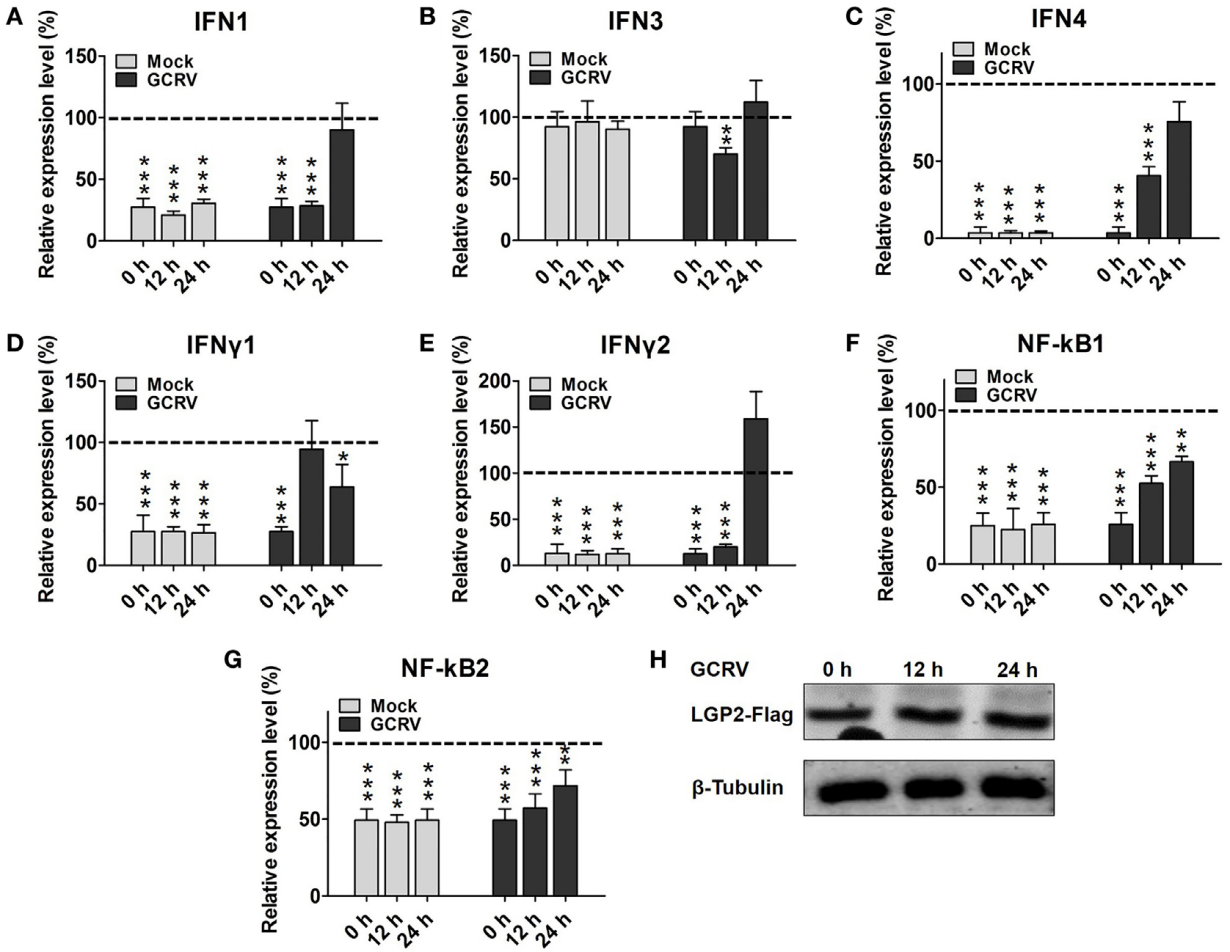
**Laboratory of genetics and physiology 2 (LGP2) overexpression decreases grass carp reovirus (GCRV)-induced IFNs and NF-κBs transcriptions at early stage**. LGP2-Flag and empty vector (pCMV-pCMV-GFP) stable transfected *Ctenopharyngodon idella* kidney (CIK) cells were seeded in 12-well plates with about 70–90% monolayer confluency. Twenty-four hours later, the cells were infected with GCRV for 12 and 24 h or time-matched mock treatment. Then the cells were harvested for qRT-PCR to quantify the relative expression levels of IFN1 **(A)**, IFN3 **(B)**, IFN4 **(C)**, IFNγ1 **(D)**, IFNγ2 **(E)**, NF-κB1 **(F)**, and NF-κB2 **(G)**, respectively. Fold change was determined relative to corresponding treatment group in empty vector (dash line). Error bars indicate significant differences from control (*0.01 < *P* < 0.05; **0.001 < *P* < 0.01; ***P < 0.001). **(H)** Analysis of LGP2-Flag post GCRV infection. LGP2-Flag stable transfected CIK cells were seeded in 6-well plates. Then the cells were treated with or without GCRV for 12 and 24 h. The cell lysate was used for WB analysis with anti-Flag and anti-β-Tubulin Abs.

**Figure 3:**
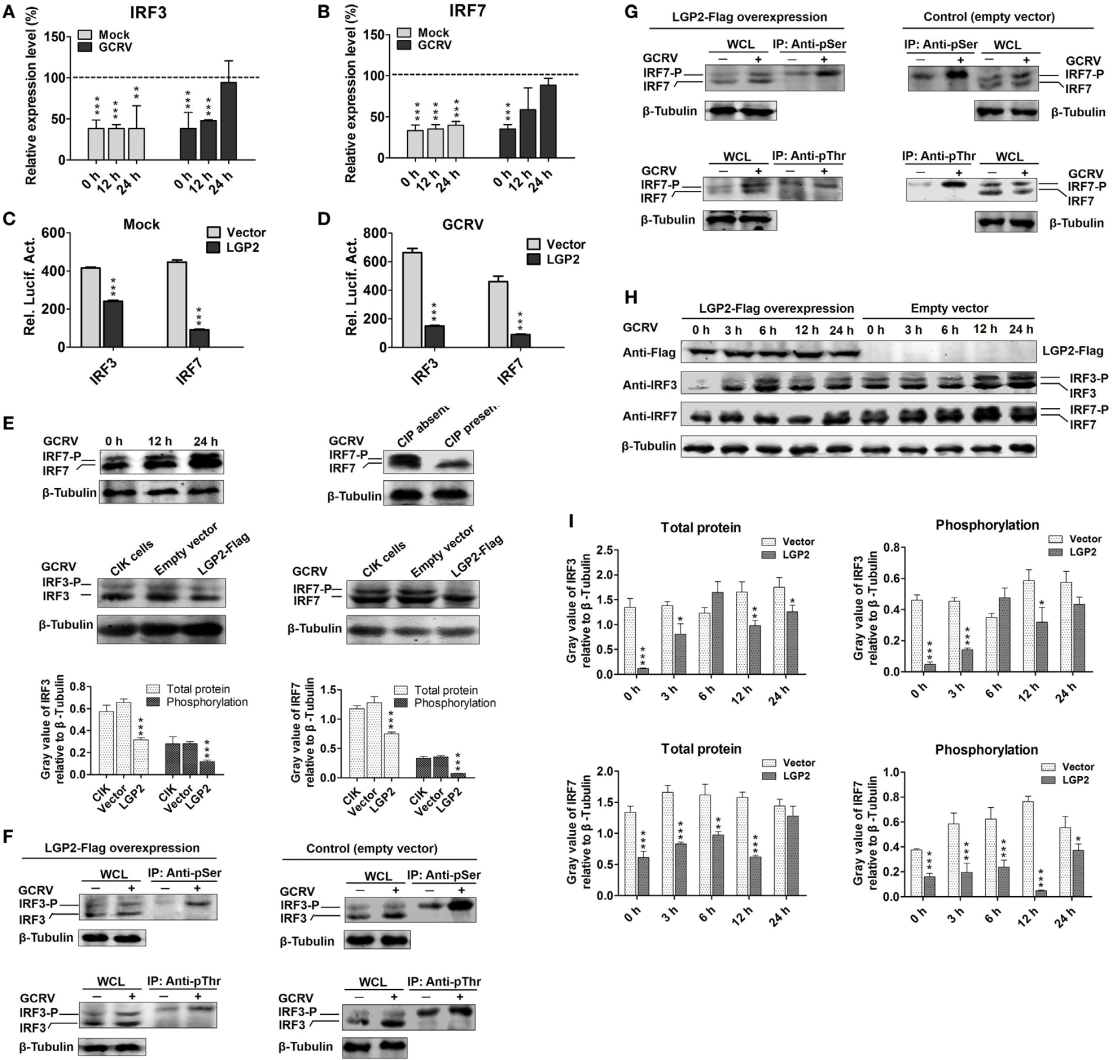
**Laboratory of genetics and physiology 2 (LGP2) suppresses interferon regulatory factor 3 (IRF3) and IRF7 signal activation *via* downregulating their synthesis and phosphorylation**. **(A,B)** LGP2 overexpression inhibits the transcriptions of IRF3 and IRF7. mRNA levels of IRF3 and IRF7 were measured by qRT-PCR in LGP2-Flag and empty vector stable transfected *C. idella* kidney (CIK) cells at 12 and 24 h post grass carp reovirus (GCRV) infection or mock treatment. Fold change was determined relative to corresponding treatment group in empty vector (dash line). Error bars indicate SD (*n* = 4). Asterisks indicate significant differences from control (***P < 0.001). **(C,D)** LGP2 overexpression inhibits the promoter activities of IRF3 and IRF7. FHM cells were cotransfected with 300 ng of LGP2-Flag, 30 ng of pRL-TK, and 300 ng of IRF3pro-luc or IRF7pro-luc plasmids. At 16 h post-transfection, the cells were infected with or without GCRV for 24 h, and then they were collected for luciferase report assay. Error bars indicate SD (*n* = 4). Asterisks indicate significant differences from control (***P < 0.001). **(E)** Upper: verification of the phosphorylation band of IRF7 antiserum. CIK cells were infected with GCRV for 12 or 24 h in 6-well plates for Western blotting (WB). A portion of the whole cell lysis (WCL), which was infected with GCRV for 24 h was incubated with or without 10 U of CIP for 30 min. IRF7 was examined by WB with IRF7 antiserum. β-Tubulin was served as an internal control. Middle: LGP2 involves in the inhibition of both IRF3 and IRF7 protein levels. CIK cells, empty vector, and LGP2-Flag-transfected cells were infected with GCRV for 12 h, and the cells lysate was used for WB. The histograms (below) display the relative expression levels, which were quantified by using ImageJ software. Error bars indicate SD (*n* = 3). Asterisks indicate significant differences from control (***P < 0.001). **(F,G)** LGP2 overexpression inhibits GCRV-induced IRF3 and IRF7 Ser and Thr phosphorylation. LGP2-Flag and empty vector stable transfected CIK cells were seeded in 10 cm^2^ dishes for GCRV infection. At 24 h post-GCRV infection, the cells were lysed for immunoprecipitation (IP) with anti-pSer and anti-pThr Abs (Ab), respectively. The IP samples and WCL were subjected to immunoblotting (IB) with anti-IRF3 and anti-IRF7 antiserum, respectively. **(H)** LGP2 overexpression represses the protein and phosphorylation levels of IRF3 and IRF7. LGP2-Flag and empty vector stable transfected CIK cells were infected with GCRV and samples were collected at 0, 3, 6, 12, and 24 h postinfection. WCL was subjected to IB with IRF3, IRF7, and β-Tubulin Ab, respectively. **(I)** The relative protein expression levels were quantified by using ImageJ software. Error bars indicate SD (*n* = 3). Asterisks indicate significant differences from control (*0.01 < *P* < 0.05; **0.001 < *P* < 0.01; ***P < 0.001).

First, we investigated the expression patterns of IFNs, NF-κBs, and IRF3/7 in mock cells (empty vector transfected cells) post-GCRV infection. The results indicate that GCRV infection significantly upregulated the mRNA levels of IFN4, IFNγ1, IFNγ2, NF-κB1, NF-κB2, IRF3, and IRF7 (Figure S2 in Supplementary Material). To explore the influence of LGP2 overexpression on IFNs and NF-κBs expressions in response to GCRV infection, LGP2-Flag stable transfected CIK cell line was infected with GCRV at different time points. qRT-PCR showed that LGP2 overexpression markedly decreases the transcriptions of IFNs and NF-κBs expect for IFN3. However, mRNA expression levels of these genes were mostly recovered to levels of control cells at 12 or 24 h after GCRV infection (Figures 2A–G). No significant change of exogenous LGP2 was detected in CIK cells upon GCRV infection (Figure 2H). Furthermore, we also examined the expressions of IRF3 and IRF7, which mediate IFN production. The results indicated that both IRF3 and IRF7 were decreased at early time points but recovered to control levels at 24 h post-GCRV infection (Figures 3A,B). These data suggested that LGP2 inhibits induction of IFNs and NF-κBs at the early phase of GCRV infection.

### LGP2 Suppresses Synthesis and Activation of IRF3 and IRF7 at the Early Phase of GCRV Infection

Interferon regulatory factor 3 and IRF7 are essential for virus-induced IFN-I activation and development of the innate antiviral responses (44). To investigate regulation of LGP2 to IRF3 and IRF7, dual-luciferase report assay was performed. As shown in Figures 3C,D, LGP2 overexpression significantly inhibits the promoter activities of IRF3 and IRF7 under both basal and GCRV infection conditions. In LGP2 overexpression CIK cells, transcription levels of IRF3 and IRF7 were notably inhibited at early stage of GCRV infection (Figures 3A,B). A previous study indicated that C-terminal phosphorylation induced by virus infection is essential for activation of IRF3 and IRF7 (44). To uncover whether LGP2 can regulate phosphorylation of IRF3 and IRF7, we first verified the recognition of phosphorylation specificity of anti-IRF7 antiserum, which has been confirmed to specifically bind recombinant IRF7 protein. CIK cells infected with GCRV at different time points (0, 12, and 24 h) were collected for WB analysis with anti-IRF7 antiserum. Two bands between 43 and 55 kDa (marker not shown) were induced by GCRV infection. The lower band was IRF7 and the upper band may be the phosphorylated form of IRF7 (Figure 3E, upper left). Then, we treated the whole cell lysis of CIK cells infected by GCRV with or without CIP. The results indicate that CIP treatment led to the disappearance of the upper band and had no influence on the basal band (Figure 3E, upper right). This result indicates that the upper band is the phosphorylated form of IRF7 indeed. Specificity and phosphorylated band of anti-IRF3 antiserum have been verified in the previous report (45). In Figure 3E, middle and below, LGP2 overexpression significantly inhibits the protein levels of IRF3 and IRF7 compared with those in CIK or empty vector-transfected cells, and empty vector has no influence on the protein expressions of IRF3 and IRF7.

Phosphorylation of the C-terminal serine (Ser) and threonine (Thr) residues is important for IRF3 and IRF7 activation following viral infection (46). To this end, IP with anti-pSer and anti-pThr Ab were performed in the LGP2-Flag stable transfected CIK cells, followed by IB with IRF3 and IRF7 antiserums, respectively. Compared with those in control (empty vector-transfected cells), LGP2 overexpression significantly inhibited GCRV-induced Ser and Thr phosphorylation of IRF3 and IRF7 (Figures 3F,G). To better understand the influence of LGP2 on the protein synthesis and activation of IRF3 and IRF7 induced by GCRV infection, LGP2-Flag and empty vector stable-transfected CIK cells were infected with GCRV at different time points. As showed in Figures 3H,I, LGP2 overexpression not only suppressed phosphorylation but also downregulated the basal protein levels of IRF3 at early phase of GCRV infection. As for IRF7, LGP2 overexpression mainly inhibited the phosphorylation levels at early time points, but had no notable effect on the basal protein levels. These results implied that LGP2 inhibits GCRV-induced activation of IRF3 and IRF7 at early phase through diverse manners: LGP2 inhibits the synthesis and Ser/Thr phosphorylation of IRF3, but mainly decreases GCRV-induced Ser/Thr phosphorylation of IRF7. Considering overall, LGP2 overexpression significantly inhibits the total protein levels (phosphorylation + basal protein) of both IRF3 and IRF7 (Figures 3H,I). We also examined tyrosine (Tyr) phosphorylation of IRF3 and IRF7. However, no Tyr phosphorylation was detected in IRF7, and IRF3 possessed Tyr residue phosphorylation but was unable to be induced by GCRV infection (Figure S3 in Supplementary Material). So, Tyr phosphorylation of IRF3 may not involve in antiviral immunity.

### Knockdown of LGP2 Enhances GCRV-Mediated Signal Induction at Early Stage

**Figure 4:**
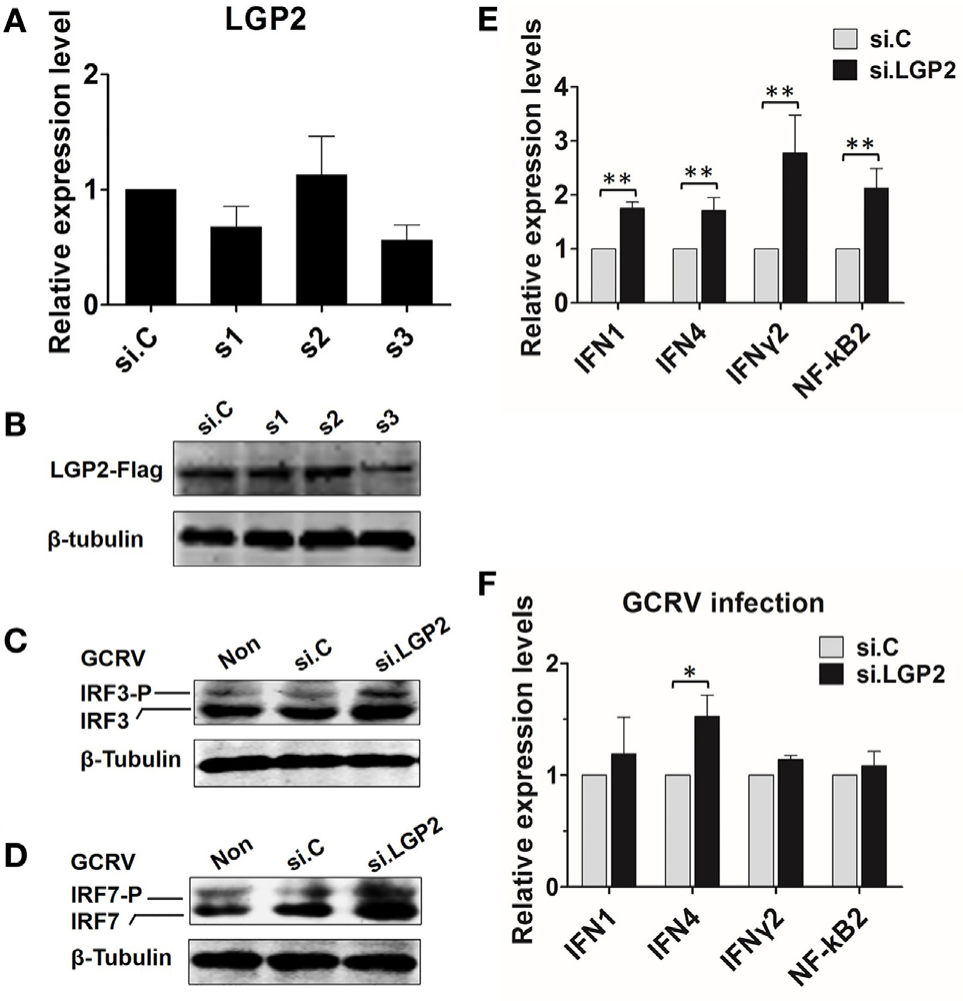
**Knockdown of laboratory of genetics and physiology 2 (LGP2) potentiates grass carp reovirus (GCRV)-mediated activation of innate immune responses in *Ctenopharyngodon idella* kidney (CIK) cells**. **(A)** Screening LGP2 interference sequences. Three siRNA sequences (s1, s2, and s3) along with the negative control si.C were transiently transfected into CIK cells. The cells were harvested for qRT-PCR at 24 h post-transfection to detect the transcription level of LGP2. **(B)** Examining the interference efficiency of the three siRNA in protein level. LGP2-Flag stable transfected CIK cells were transiently transfected with s1, s2, s3, and si.C in 6-well plates. Twenty-four hours later, cell lysates were prepared for IB using anti-Flag Ab. **(C,D)** Knockdown of LGP2 upregulates the protein levels of IRF3 and IRF7 induced by GCRV infection. CIK cells were transfected with s3 in 6-well plates. Twelve hours later, cells were infected with GCRV for 12 h and WB was conducted with anti-IRF3 and anti-IRF7 antiserums, respectively. **(E,F)** CIK cells were transfected with s3 and si.C, respectively, and treated or untreated with GCRV for 12 h. The cells were prepared for qRT-PCR to test the transcription levels of IFN1, IFN4, IFNγ2, and NF-κB2, respectively. Error bars indicate SD (*n* = 4). Asterisks indicate significant differences from control (*0.01 < *P* < 0.05; **0.001 < *P* < 0.01).

To verify the results obtained from above experiments, LGP2 in CIK cells (endogenous) or in LGP2 stable overexpression CIK cells was silenced by LGP2-specific siRNA. Among three candidate siRNA sequences, s3 showed the best interference efficiency in LGP2 mRNA level in CIK cells (Figure 4A). Consistently, s3 induced significant knockdown in LGP2 protein level in LGP2 stable overexpression CIK cells (Figure 4B), so, s3 was selected for the following experiments. Compared with the untransfected or transfected with control siRNA, LGP2 knockdown significantly increased GCRV-induced basal protein and phosphorylation levels of IRF3 and IRF7 (Figures 4C,D). To further detect the influence of LGP2 knockdown on virus-triggered immune genes, IFN1, IFN4, IFNγ2, and NF-κB2, which were chosen as representations for IFNs and NF-κBs, respectively, were examined by qRT-PCR. Inversely correlated with the results in Figure 2, knockdown of LGP2 remarkably upregulated the basal inductions of these genes (Figure 4E). However, upon GCRV infection, mRNA expressions of these genes showed a trend to recover to control levels (Figure 4F). These results further confirm the negative role of LGP2 in antiviral immune responses at early stage.

### LGP2 Interacts with RIG-I and MDA5 Independent of GCRV Infection

**Figure 5:**
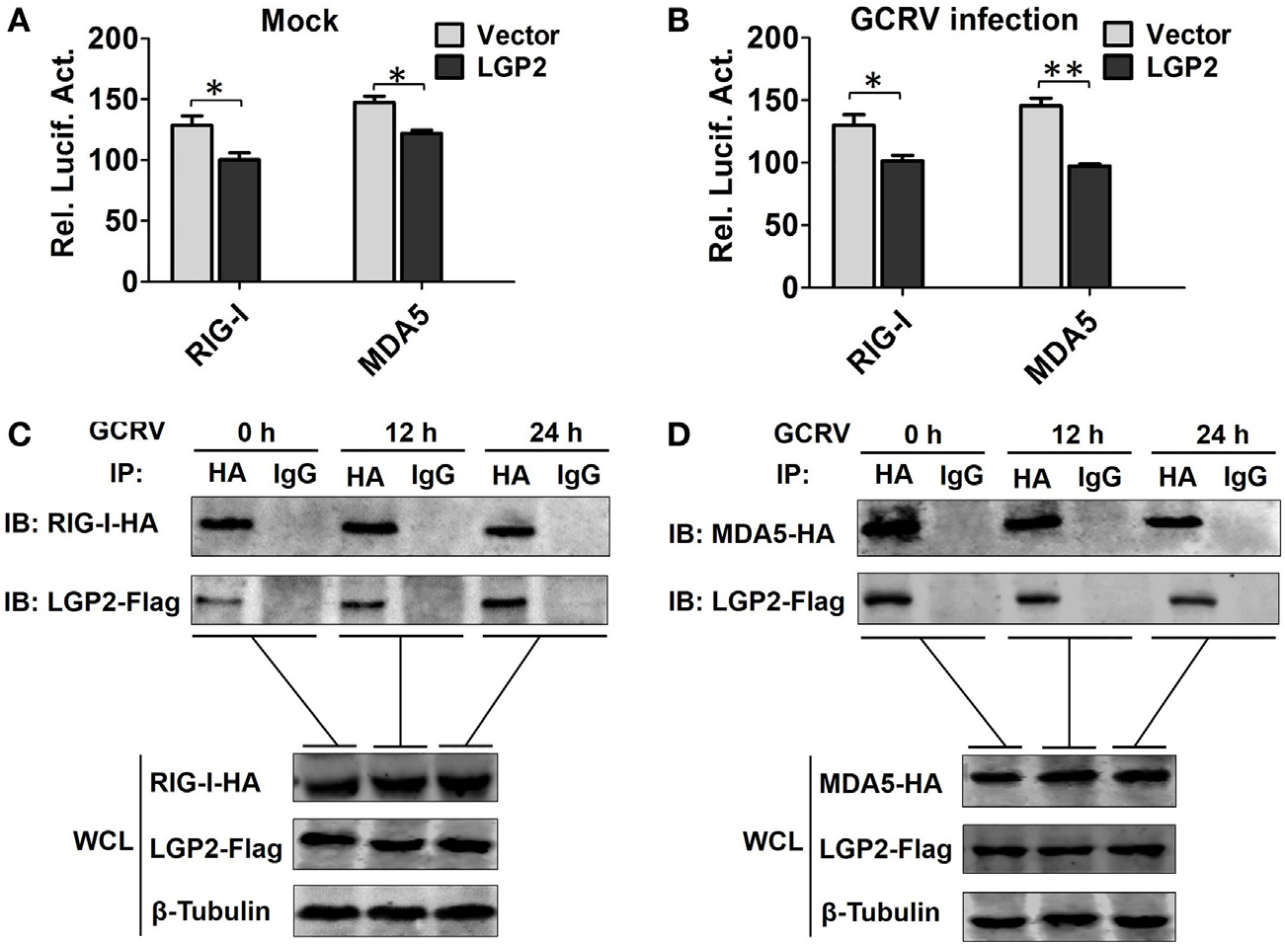
**Laboratory of genetics and physiology 2 (LGP2) interacts with melanoma differentiation-associated gene 5 (MDA5) and retinoic acid-inducible gene I (RIG-I) independent of grass carp reovirus (GCRV) infection**. **(A,B)** LGP2 overexpression inhibits RIG-I and MDA5 promoter activities. Fathead minnow (FHM) cells were transiently transfected with 300 ng of LGP2-Flag overexpression plasmid or empty vector, 30 ng of pRL-TK, and 300 ng of report vector (RIG-Ipro-luc or MDA5pro-luc) for 16 h, and then the cells were infected with GCRV or uninfected. Dual-luciferase report assays were conducted at 24 h after GCRV infection. Error bars indicate SD (*n* = 4). Asterisks indicate significant differences from control (*0.01 < *P* < 0.05; **0.001 < *P* < 0.01). **(C,D)** LGP2 interacts with RIG-I and MDA5. FHM cells were transfected with LGP2-Flag and RIG-I-HA or LGP2-Flag and MDA5-HA for 16 h, and then infected with GCRV for 12 or 24 h. Co-IP was performed with anti-HA monoclonal antibody (Ab). Mouse IgG was used as control. WCL of each time point was subjected to IBs with anti-Flag, anti-HA, and β-Tubulin Ab, respectively.

A previous report indicated that LGP2 functions upstream of RIG-I and MDA5 (18). Consistently, LGP2 overexpression significantly inhibited the promoter activities of RIG-I and MDA5 (Figures 5A,B). To determine whether LGP2 can directly interact with RIG-I or MDA5, flag-tagged LGP2 was co-transfected with HA-tagged RIG-I or MDA5 into FHM cells. Co-IP assay was carried out with anti-HA, and IB analysis was performed with anti-HA or anti-Flag Ab. As showed in Figures 5C,D, LGP2 efficiently interacted with RIG-I and MDA5 no matter under basal condition or GCRV infection. Meanwhile, similar results were obtained from the reverse Co-IP assay (Figure S4 in Supplementary Material). These results demonstrate that LGP2 interact with RIG-I and MDA5 independent of GCRV infection.

### LGP2 Restrains RIG-I- and MDA5-Mediated IPS-1 and MITA Activation

**Figure 6:**
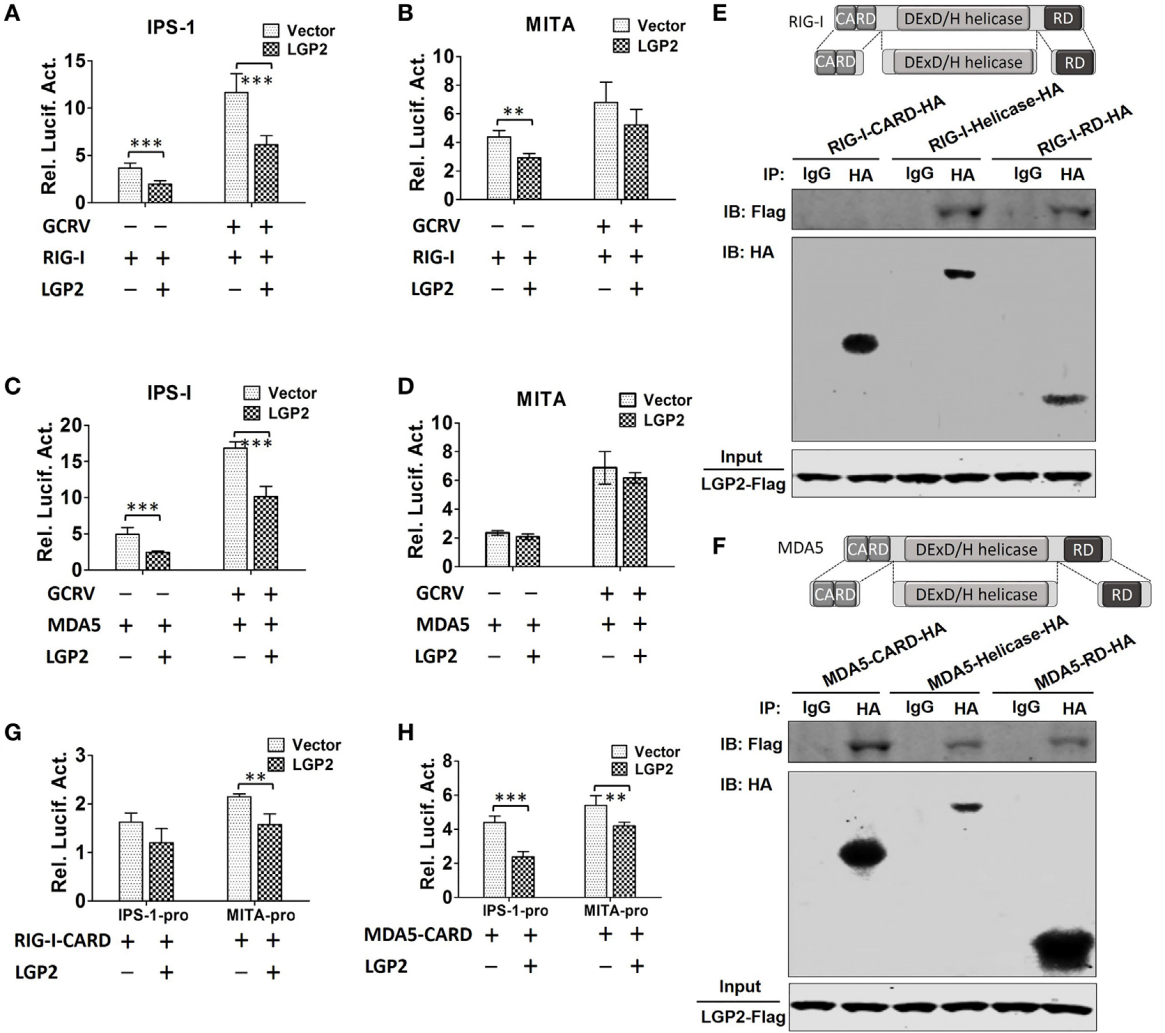
**Laboratory of genetics and physiology 2 (LGP2) inhibits downstream signaling of retinoic acid-inducible gene I (RIG-I) and melanoma differentiation-associated gene 5 (MDA5)**. **(A,B)** LGP2 inhibits RIG-I-mediated IFN-β promoter stimulator 1 (IPS-1) and mediator of IRF3 activation (MITA) promoter activities. Fathead minnow (FHM) cells were transiently transfected with 200 ng of LGP2-Flag or empty vector, 200 ng of RIG-I expression plasmid, 30 ng of pRL-TK plus 200 ng of IPS-1pro-luc or MITApro-luc for 16 h and then infected with grass carp reovirus (GCRV). Luciferase activities were conducted at 24 h after GCRV infection. **(C,D)** LGP2 inhibits MDA5-mediated IPS-1, but not MITA promoter activity. FHM cells were transiently transfected with 200 ng of LGP2-Flag or empty vector, 200 ng of MDA5 expression plasmids, 30 ng of pRL-TK plus 200 ng of IPS-1pro-luc or MITApro-luc in 24-well plates. At 16 h post-transfection, the cells were infected with GCRV for 24 h and then subjected to luciferase activities analysis. **(E)** Upper: schematic representations of full-length RIG-I and the three domains constructed in the present study. Below: LGP2 interacts with RIG-I-Helicase, RIG-I-RD, but not RIG-I-CARDs domain. FHM cells were cotransfected with 4 µg LGP2-Flag and 4 µg RIG-I-CARD-HA or RIG-I-Helicase-HA or RIG-I-RD-HA for 24 h in 10 cm^2^ dishes. Co-IP was performed using anti-HA antibody (Ab), and mouse IgG was used as control. IPs were analyzed by IBs with anti-HA and anti-Flag, respectively. Expression of LGP2-Flag (input) was examined with anti-Flag. **(F)** Upper: full-length MDA5 and its domain structures. Below: LGP2 interacts with MDA5-CARDs, MDA5-Helicase, and MDA5-RD. FHM cells were transfected with the indicated plasmids (4 µg each). Twenty-four hours later, cells were lysed, Co-IP and IB analyses were performed with the indicated Abs. **(G)** LGP2 inhibits RIG-I-CARDs-mediated MITA, but not IPS-1 promoter activity. FHM cells were transfected with 200 ng of LGP2-Flag or empty vector, 200 ng of RIG-I-CARD-HA expression plasmid, 30 ng of pRL-TK plus 200 ng of IPS-1pro-luc or MITApro-luc. Luciferase assays were performed at 24 h post-transfection. **(H)** LGP2 inhibits MDA5-CARDs-mediated IPS-1 and MITA promoter activities. FHM cells were transfected with 200 ng of LGP2-Flag or empty vector, 200 ng of MDA5-CARD-HA expression plasmid, 30 ng of pRL-TK plus 200 ng of IPS-1pro-luc or MITApro-luc. Luciferase assays were performed at 24 h post-transfection. Error bars indicate SD (*n* = 4). Asterisks indicate significant differences from control (**0.001 < *P* < 0.01; ***P < 0.001).

Upon activation, RIG-I and MDA5 induce downstream signaling *via* interaction with IPS-1 and MITA (10, 20). To determine whether LGP2 can restrain RIG-I-, MDA5-mediated IPS-1, MITA promoter activities upon GCRV infection, dual-luciferase reporter assays were performed in FHM cells. The results indicate that LGP2 overexpression inhibits RIG-I- and MDA5-mediated basal and GCRV-induced IPS-1 promoter activities (Figures 6A,C). However, LGP2 overexpression just inhibits RIG-I-mediated basal, but not GCRV-triggered activity of MITA promoter, and had no influence on MDA5-mediated MITA promoter activity (Figures 6B,D). As well known, RIG-I and MDA5 activate IPS-1 *via* a CARD–CARD-mediated interaction (1). To identify the domain specificity of the interaction between LGP2 and RIG-I or MDA5, HA-tagged RIG-I or MDA5 domains (CARDs, helicase, and RD) expression plasmids were constructed (Figures 6E,F upper). IP assay indicates that LGP2 specifically interacts with RIG-I helicase and RD domains, but not the CARDs domain (Figure 6E below). However, LGP2 interacts with all the three domains of MDA5 (Figure 6F, below). These observations imply the difference of LGP2 in regulating RIG-I and MDA5. Do the different interactions of LGP2 with RIG-I or MDA5 CARDs domain affect IPS-1 activation? To this end, we tested the influence of LGP2 on the promoter activities of IPS-1 and MITA mediated by RIG-I CARDs or MDA5 CARDs. Interestingly, LGP2 overexpression suppresses both RIG-I CARDs- and MDA5 CARDs-mediated MITA promoter activities (Figures 6G,H). For IPS-1 promoter, LGP2 overexpression significantly inhibits MDA5 CARDs-mediated promoter activity of IPS-1 (*P* < 0.001), but had no notable inhibition in RIG-I CARDs-mediated IPS-1 activity (*P* > 0.05) (Figures 6G,H). These results collectively demonstrate that LGP2 inhibits RLRs signaling *via* direct protein–protein interaction with RIG-I and MDA5. Difference in interaction of LGP2 with RIG-I CARDs or MDA5 CARDs also implies the distinguishable regulation strategies of LGP2 to RIG-I and MDA5.

### LGP2 Inhibits K63-Linked Ubiquitination of RIG-I and MDA5

**Figure 7:**
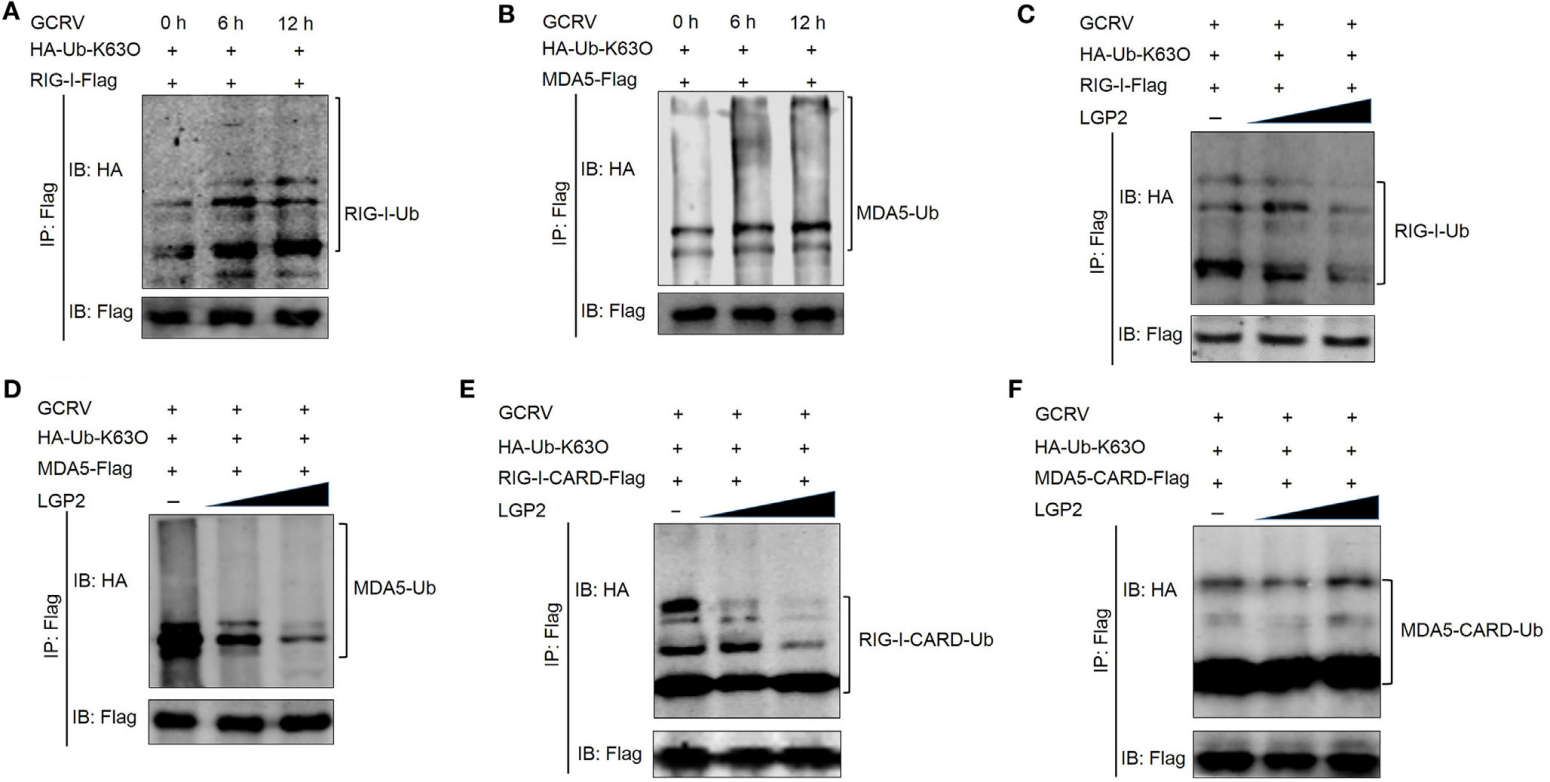
**Laboratory of genetics and physiology 2 (LGP2) inhibits the K63-linked ubiquitination of retinoic acid-inducible gene I (RIG-I) and melanoma differentiation-associated gene 5 (MDA5)**. **(A,B)** Grass carp reovirus (GCRV) infection upregulates the K63-linked ubiquitination of RIG-I and MDA5. Fathead minnow (FHM) cells were transfected with 2 µg HA-Ub-K63O, 6 µg RIG-I-Flag **(A)**, or 6 µg MDA5-Flag **(B)** in 10 cm^2^ dishes, respectively. At 24 h post-transfection, the cells were treated with MG132 and GCRV for 6 and 12 h. Then, the cells were harvested for immunoprecipitation (IP) with anti-Flag antibody (Ab) and IBs with anti-HA and anti-Flag Ab. **(C,D)** LGP2 inhibits the K63-linked ubiquitination of RIG-I and MDA5 in a dose-dependent manner. FHM cells were seeded in 10 cm^2^ dishes for 24 h and transfected with 1 µg HA-Ub-K63O, LGP2 (0, 1.5, and 3 µg) together with decreasing amounts of empty vector (3, 1.5, and 0 µg), 4 µg RIG-I-Flag **(C)**, or 4 µg MDA5-Flag **(D)**. At 24 h post-transfection, the cells were treated with MG132 and GCRV for 6 h. Then, the cells were harvested for IP with anti-Flag Ab and IBs with anti-HA and anti-Flag Ab, respectively. **(E,F)** LGP2 represses the K63-linked ubiquitination of RIG-I CARDs, but not MDA5 CARDs. FHM cells were transfected with 1 µg HA-Ub-K63O, LGP2 (0, 1.5, and 3 µg), empty vector (3, 1.5, and 0 µg), 4 µg RIG-I-CARD-Flag **(E)**, or 4 µg MDA5-CARD-Flag **(F)** in 10 cm^2^ dishes. At 24 h post-transfection, the cells were treated with MG132 and GCRV for 6 h. Then, the cells were harvested for IP with anti-Flag Ab and IBs with the indicated Abs.

Previous studies have demonstrated that K63-linked ubiquitination positively regulates downstream signaling of RIG-I and MDA5 in antiviral innate immune responses (6, 47). Given the negative regulation of LGP2 in RIG-I- and MDA5-mediated downstream signaling, whether LGP2 can affect the K63-linked ubiquitination of RIG-I and MDA5? To this end, *in vivo* ubiquitination assay was performed in FHM cells. Our results indicated that GCRV infection enhanced the K63-linked ubiquitination of both RIG-I and MDA5 (Figures 7A,B). Meanwhile, LGP2 overexpression inhibited the K63-linked ubiquitination of both RIG-I and MDA5 in a dose-dependent manner (Figures 7C,D). In mammals, binding to K63 ubiquitin chain in CARDs domain is essential for activation of RIG-I and MDA5 (47). In order to gain more insights into the impact of LGP2 on RIG-I and MDA5 activation, K63-linked ubiquitination of RIG-I CARDs and MDA5 CARDs were further examined. Interestingly, LGP2 overexpression significantly inhibited the K63-linked ubiquitination of RIG-I CARDs but had no influence on the ubiquitination of MDA5 CARDs (Figures 7E,F). These results indicate that LGP2 represses MDA5 activation by way of inhibiting the K63-linked ubiquitination of MDA5 helicase or RD domains, but not CARDs. However, suppression of RIG-I CARDs K63-linked ubiquitination is important for LGP2-inhibited activation of RIG-I. Sequence alignment indicated that K154, K164, K169, and K172 residues, which bind K63-linked polyubiquitin chain are conserved in grass carp RIG-I CARDs (Figure S5A in Supplementary Material) (48–50), so the ability to bind K63 polyubiquitin chain of grass carp RIG-I CARDs is similar with that in mammals.

### LGP2 Suppresses K48-Linked Ubiquitination of RIG-I and MDA5 at Early Stage during GCRV Infection

**Figure 8:**
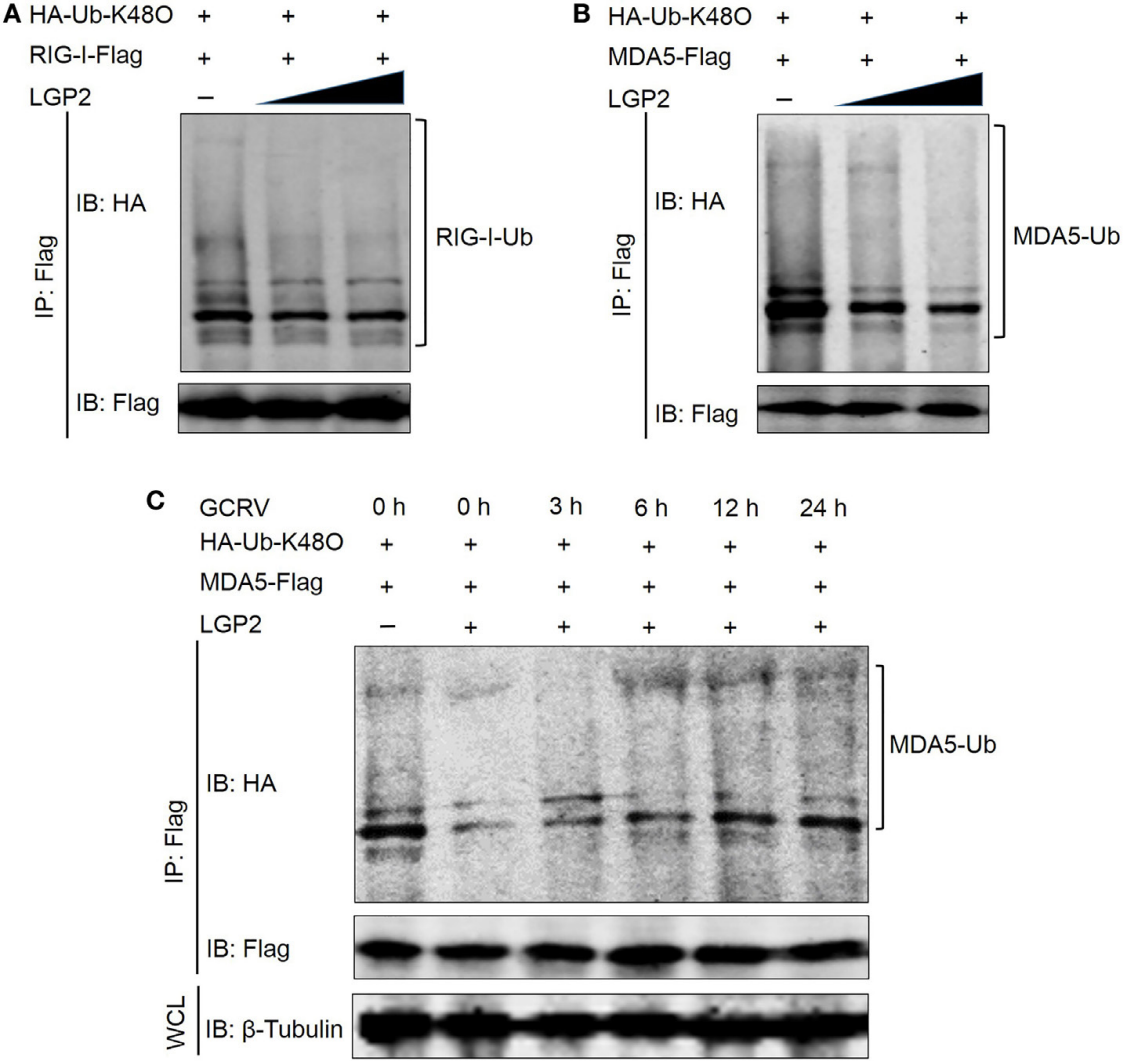
**Laboratory of genetics and physiology 2 (LGP2) mediates the K48-linked ubiquitination of retinoic acid-inducible gene I (RIG-I) and melanoma differentiation-associated gene 5 (MDA5)**. **(A,B)** LGP2 significantly inhibits the K48-linked ubiquitination of MDA5 but slightly reduces that of RIG-I. Fathead minnow (FHM) cells were transfected with 1 µg HA-Ub-K48O, LGP2 (0, 1.5, and 3 µg) together with decreasing amounts of empty vector (3, 1.5, and 0 µg), 4 µg RIG-I-Flag **(A)**, or 4 µg MDA5-Flag **(B)**. At 24 h post-transfection, the cells were treated with MG132 for 6 h, and the cells were harvested for immunoprecipitation (IP) with anti-Flag antibody (Ab) and IBs with anti-HA and anti-Flag Ab, respectively. **(C)** LGP2 suppresses the K48-linked ubiquitination of MDA5 at early stage of grass carp reovirus (GCRV) infection. FHM cells were transfected with 1 µg HA-Ub-K48O, 2 µg LGP2, 1 µg empty vector, and 4 µg MDA5-Flag. Control cells were transfected with 1 µg HA-Ub-K48O, 3 µg empty vector, and 4 µg MDA5-Flag. At 24 h post-transfection, the cells were infected with GCRV for 3, 6, 12, and 24 h and treated with MG132 for 6 h. After that, the cells were lysed and subjected to IP with Flag Ab and subsequent IB with anti-HA and anti-Flag, respectively. WCL was used for IB with anti-β-Tubulin.

To investigate whether LGP2 is involved in the proteasome-mediated degradation of RIG-I and MDA5, the K48-linked ubiquitination of RIG-I and MDA5 was examined upon LGP2 overexpression. Surprisingly, LGP2 overexpression did not promote the degradation of RIG-I and MDA5, but significantly depressed the K48-linked ubiquitination of MDA5 in a dose-dependent manner and slightly inhibited that of RIG-I at steady state (Figures 8A,B). These results raise a question that what is the biological significances of LGP2-triggered inhibition of RIG-I and MDA5 degradation? Then, MDA5 was selected for further examination of the K48-linked ubiquitination at different time points post-GCRV infection. Comparatively, the K48-linked ubiquitination of MDA5 was significantly inhibited by LGP2 overexpression at early phase of GCRV infection, but gradually recovered to the control level at later time (Figure 8C). These results collectively demonstrate that LGP2-induced inhibition of MDA5 and RIG-I K48-linked ubiquitination just occurs at resting state and early stage post-GCRV infection.

## Discussion

In contrast to previous reports of LGP2 as a positive regulator of MDA5- and RIG-I-mediated viral recognition (16, 18, 51), our present study demonstrates that grass carp LGP2 is a negative regulator in RIG-I- and MDA5-mediated antiviral signaling pathway at resting state and early phase during GCRV infection. Previous investigations found the negatively regulatory role of LGP2 in IFN signaling: overexpression of LGP2 strongly inhibits IRF3 activation and IFN-stimulated regulatory element and NF-κB signaling pathways post-Newcastle disease virus infection (13). LGP2 can inhibit antiviral signaling independent of dsRNA or virus infection (15). IRF family has been demonstrated to contain 9 members in mammals, 10 members in avian, and 13 members in fish (43). IRF3 and IRF7, two structurally homologous members, are able to activate fish IFN promoters and upregulate fish IFNs and ISGs (52). Here, the inhibition of IRF3, IRF7, IFNs, and NF-κBs promoter activities and mRNA expression levels caused by LGP2 overexpression provides direct proofs for the negative role of LGP2. This conclusion is also verified by LGP2 knockdown assay.

In the resting state cells, IRF3 and IRF7 localize in cytoplasm, whereas poly(I:C) stimulation or virus infection induces their cytoplasmic-nuclear translocation (20, 45). Phosphorylation is the prerequisite for activation and nuclear import of IRF3 and IRF7. In line with the result from luciferase report assays, LGP2 overexpression inhibits both Ser and Thr phosphorylation of IRF3 and IRF7, which is the substance that LGP2 suppresses activation of IRF3 and IRF7. IRF3 and IRF7 are proposed to synergistically induce the expressions of IFNs (52). However, compared with these abundant data about the relationship between LGP2 and IRF3, no evidence reflects regulation model of LGP2 to IRF7 (13). Our results first identified the negative regulation of LGP2 to IRF7 in the promoter activity, mRNA, and protein level in response to dsRNA virus infection in fish cells. We also revealed the different mechanisms of LGP2 in regulating IRF3 and IRF7: in resting state and early stage of GCRV infection, LGP2 overexpression inhibits both basal protein and phosphorylation levels of IRF3 but just reduces the phosphorylation level of IRF7.

**Figure 9:**
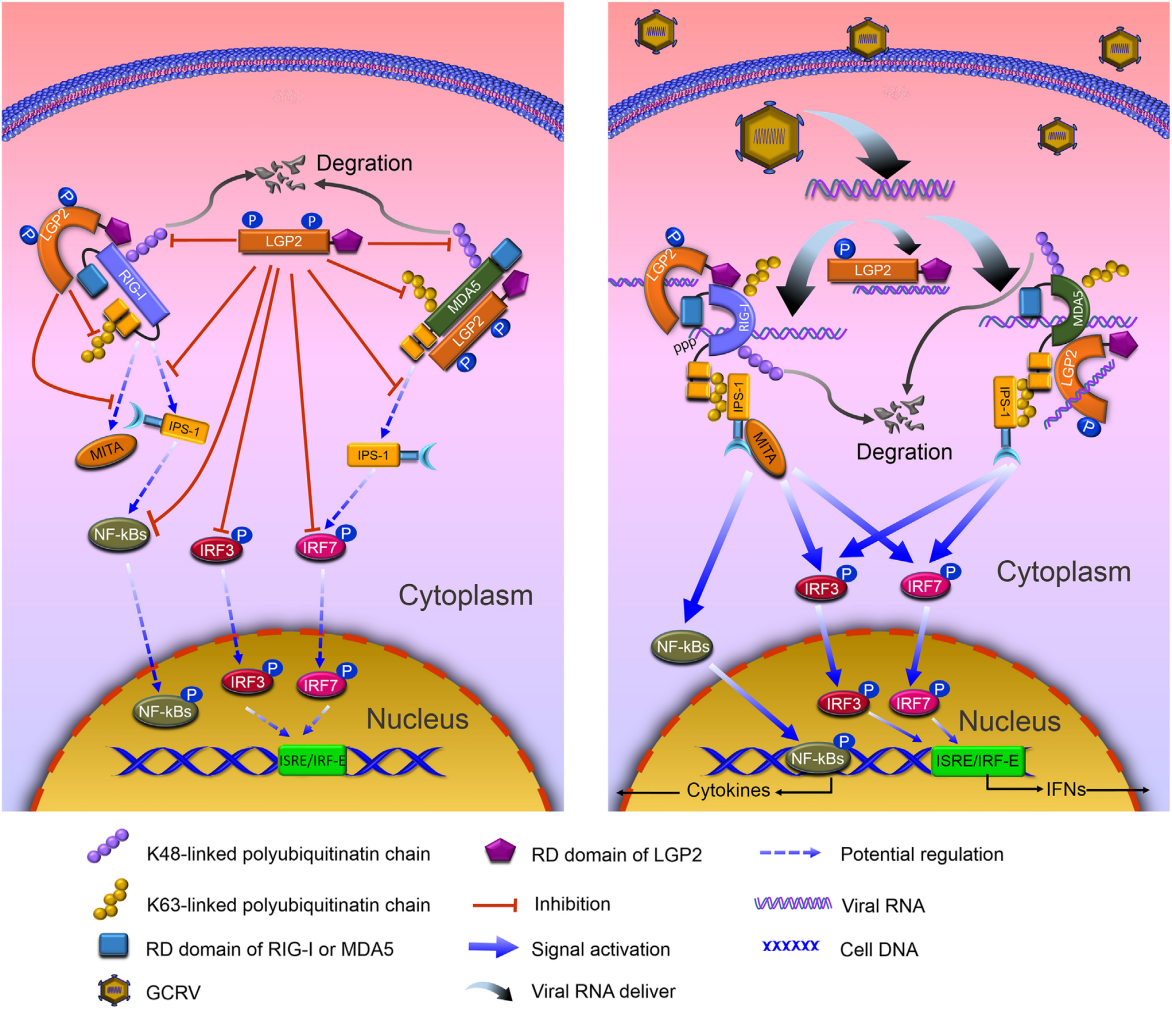
**Model of negative role of laboratory of genetics and physiology 2 (LGP2) in modulating retinoic acid-inducible gene I (RIG-I)- and melanoma differentiation-associated gene 5 (MDA5)-mediated antiviral signaling in grass carp**. Left: in resting state, LGP2 binds Helicase and repressor domains (RDs) of RIG-I, but leaves the CARDs to form an anti-inhibited state with Helicase, which weakens binding with downstream adaptor IFN-β promoter stimulator 1 (IPS-1). Besides binding to Helicase and CARDs domains, LGP2 competitively interacts with the CARDs of MDA5 that represses MDA5 to form anti-inhibited comformation and interaction with IPS-1. Meanwhile, LGP2 suppresses K63-linked polyubiquitination of RIG-I CARDs and MDA5 Helcase or RD domains. Consequently, signal transductions from RIG-I and MDA5 to IPS-1 and mediator of IRF3 activation (MITA) are inhibited. Furthermore, LGP2 restrains phosphorylation and expressions of IRF3 and IRF7, and the subsequent signals of NF-κBs and IFNs. Concomitantly, LGP2 suppresses the degradation of RIG-I and MDA5 through inhibiting K48-linked polyubiquitination of RIG-I and MDA5, which ensures the basal levels of RIG-I and MDA5 for subsequent antiviral activation. Right: grass carp reovirus (GCRV) infection induces the activation of RLR signals, yet vanishes LGP2-induced inhibiton. dsRNAs derived from GCRV facilitate conformational change of RIG-I and MDA5. Activated RIG-I and MDA5 release the CARDs, which interact with the CARD of IPS-1. IPS-1, then associates with MITA, and activates downstream signals *via* NF-κBs and IRF3/IRF7-IFNs pathways. However, in GCRV invading cells, LGP2 remains to interact with RIG-I and MDA5, disappears Thr-phosphorylation, which may contribute to the derepression for RLR-mediated activation.

In Huh7 cells, Saito et al. found that LGP2 overexpression can form a stable complex with RIG-I or MDA5 (14). Our Co-IP experiments demonstrated that LGP2 interacts with RIG-I or MDA5 independent of GCRV infection. Saito et al. also described that LGP2 just represses RIG-I signaling, but is not sufficient for MDA5 signaling inhibition (14). However, the direct interactions of grass carp LGP2 with RIG-I or MDA5 functionally inhibit RIG-I- or MDA5-induced IPS-1 promoter activities. Classical model supports that RIG-I adopts a closed autoinhibited conformation where CARDs are sterically masked and unavailable for signal transduction in resting cells (1). In our domain interaction assay, no direct interaction was observed between LGP2 and RIG-I CARDs, meanwhile, LGP2 fails to block RIG-I CARDs-mediated promoter activity of IPS-1 at resting state. These results indicate that LGP2 inhibits RIG-I-mediated signal transduction independent of direct binding with RIG-I CARDs, which is in line with the previous report that LGP2 controls RIG-I signaling through *in trans* interaction between RIG-I helicase domain and LGP2 RD domain (14). The present study proposes the following model of LGP2 in mediating RIG-I signaling: under normal condition, LGP2 invertedly binds to RIG-I (LGP2 helicase domain interacts with RIG-I RD and LGP2 RD binds to RIG-I helicase domain); upon viral infection, cooperative ATP and viral dsRNA binding to RIG-I helicase domain leads to a conformational switch to a closed form with dsRNA, and the CARDs are released to interact with IPS-1 concomitantly (53). Unlike RIG-I, MDA5 is thought to adopt an open conformation with exposed CARDs in the absence of ligand (1). Our interaction study provided an efficient interaction between LGP2 and MDA5 CARDs. Importantly, LGP2 indeed significantly represses MDA5 CARDs-mediated IPS-1 promoter activity (Figure 6H). A reasonable mechanism may be that strong interaction between LGP2 and MDA5 CARDs inhibits MDA5 to establish an intramolecular interaction. In other words, LGP2 binds to MDA5 CARDs, which fails to make MDA5 form a self-inhibited state. Simultaneously, this interaction efficiently restrains MDA5 CARDs-mediated signaling to IPS-1 (Figure 9). The exact interaction relationship between domains of LGP2 and MDA5 are still unknown. Comparatively, LGP2 shows more preference to restrain RIG-I- rather than MDA5-modulated MITA promoter activity. In zebrafish, MITA associates with RIG-I–IPS-1 complexes, but not with that involving MDA5–IPS-1. It is likely that fish MITA is a key scaffolding protein of RIG-I rather than MDA5 (8). However, in some RIG-I-null species, such as chicken and Chinese tree shrew, MITA can interact with MDA5 to mediate the corresponding signaling. Knockdown of MITA inhibits MDA5-mediated IFN-β activation (9, 19). Therefore, experimental evidence needs to be proposed to identify whether MITA is essential for MDA5 signaling pathway in RIG-I-existed species and compare the difference between RIG-I- and MDA5-mediated MITA downstream signals.

The ubiquitin system is responsible for regulating almost all the host cellular processes. Numerous studies have highlighted the important insights into the regulation of protein stability, immune activation, and host–pathogen interplay by protein ubiquitination (1, 54, 55). In the present study, LGP2 overexpression significantly inhibited the K63-linked ubiquitination of full-length RIG-I and MDA5. But for CARDs domain, LGP2 just suppressed the K63-induced ubiquitination in RIG-I, not in MDA5. These results suggest that LGP2 utilizes different mechanisms to modulate the K63-linked ubiquitination of RIG-I and MDA5. Tripartite motif 25 (TRIM25, also called Efp) and Riplet (also called Reul or RNF135) are two important E3 ubiquitin ligases for the K63-linked ubiquitination of RIG-I (56). A study has demonstrated that the K63-linked ubiquitination mediated by TRIM25 in K172 residue in RIG-I CARDs is indispensable for IPS-1 recruitment (57). Meanwhile, K154, K164, and K172 residues of RIG-I CARDs are critical for Riplet-mediated K63-linked ubiquitination and antiviral signal transduction of RIG-I (48). Interestingly, these residues are conserved in grass carp RIG-I (Figure S5A in Supplementary Material). In all probability, LGP2 inhibits the K63-linked ubiquitination of RIG-I through regulating these ubiquitin E3 ligases. For MDA5, even though the present study has demonstrated that MDA5 CARDs can bind to K63 ubiquitin chain, the specific ubiquitin ligase and residue remain unresolved (47). LGP2 restrains the K63-linked ubiquitination of MDA5 independent of CARDs domain.

Unlike the K63-linked ubiquitination, the K48-conjugated ubiquitination chain delivers the substrates to the proteasomes for degradation. A study has demonstrated that RNF125, an E2 ubiquitin-conjugating enzyme, mediates the degradation of RIG-I and signaling impairment of MDA5 *via* the K48-linked ubiquitination (56). Surprisingly, grass carp LGP2 did not promote but inhibited the K48-linked ubiquitination, especially for MDA5, which suggests that LGP2 functions as a “positive” regulator for RIG-I and MDA5. For these seemingly contradictory results, an optimal interpretation may be that: at resting state and early phase of virus invasion, LGP2, on one hand, restrains the K63-linked ubiquitination of RIG-I and MDA5 to inactivate downstream signaling, on the other hand, inhibits the K48-linked ubiquitination to suppress RIG-I and MDA5 degradation to guarantee the basal protein levels, which are crucial for subsequently rapid signal activation. Our subsequent results further supported this hypothesis that upon GCRV infection, the K48-linked ubiquitination of MDA5 induced by LGP2 gradually recovers to normal level in time-dependent manner. As we know, uncontrolled antiviral responses have deleterious effects on the host (52). Therefore, to control excessive immune responses and maintain cellular homeostasis, LGP2 may function as a balancer for RLR signal transduction: under resting state, make immune system “keep silence” and “activate rapidly” upon virus infection (Figure 9). Interactions between LGP2 and RIG-I or MDA5 are independent of GCRV infection. How does LGP2 transform its function from inhibition to derepression? Possible explanation may owe to binding viral RNA, which results in modification change of LGP2. Our study found that LGP2 possesses Thr and Tyr phosphorylation and GCRV infection leads to dephosphorylation of LGP2 Thr residue (Figure S6 in Supplementary Material). Probably, phosphorylation is involved in the regulation of LGP2 function. In addition, sequence of fish LGP2 holds low similarity with mammalian, although LGP2 is structurally conserved in vertebrate (Figure S5B in Supplementary Material). Therefore, fish LGP2 may possess peculiar modifications, which are different from those in mammals.

In conclusion, our findings provide novel insights into the negative role of LGP2 in RLR signal modulation. Grass carp LGP2 directly interacts with RIG-I and MDA5 and suppresses downstream signal activations of IPS-1 and MITA *via* dual regulations of RIG-I and MDA5 by the K48- and K63-linked ubiquitination, then represses expressions and phosphorylation of IRF3 and IRF7. All of these finally inhibit productions of IFNs and NF-κBs. Upon GCRV infection, LGP2, first, undergoes gradual disinhibition and then allows the robust antiviral immune responses (Figure 9). However, additionally, experimental proofs are required to illuminate the precise mechanisms of LGP2 in regulation of RIG-I and MDA5 ubiquitination, and the essential role of LGP2 Thr phosphorylation in its functional regulation.

## Author Contributions

JS and YR conceived and designed the experiments. YR, QW, and CY performed the experiments and analyzed these data. YR and JS wrote the manuscript. All authors reviewed the manuscript.

## Conflict of Interest Statement

The authors declare that the research was conducted in the absence of any commercial or financial relationships that could be construed as a potential conflict of interest.

## Footnotes

^1^http://www-bimas.cit.nih.gov/molbio/proscan/.

^2^http://gpminer.mbc.nctu.edu.tw/index.php.

^3^http://www.cbs.dtu.dk/services/Promoter/.
