# Supplementary material for: Grass Carp Laboratory of Genetics and Physiology 2 Serves As a Negative Regulator in Retinoic Acid-Inducible Gene I- and Melanoma Differentiation-Associated Gene 5-Mediated Antiviral Signaling in Resting State and Early Stage of Grass Carp Reovirus Infection
Source: Front Immunol. 2017 Mar 27;8:352. doi: 10.3389/fimmu.2017.00352 (PMC5366347; doi:10.3389/fimmu.2017.00352)
Supplement: Supplementary file 1 [file Data_Sheet_1.DOCX]

**Supporting Information**

**Supplemental Table 1**

Primer sequences and their designated applications in the plasmid vector construction.

| Gene name | Plasmid name | Primer name | Primer sequence (5'–3') |
| --- | --- | --- | --- |
| RIG-I | RIG-Ipro-luc | RF262 | **ACTG**ctcgagTTGAAGTTATCATCATCTACAGTGC |
|  |  | RR263 | **ACTG**aagcttACTACAAACAAGGAAACGAAAGA |
| MDA5 | MDA5pro-luc | MF264 | **ACTG**ctcgagTAATTTTGTTATACGCTAAAAAAGAT |
|  |  | MR265 | **ACTG**aagcttGATAAGAGACTAGATGAGAAAAGTTC |
| IPS-1 | IPS-1pro-luc | IF405 | **ACTG**ctcgagAACCAGTCAGCGTGGGAAT |
|  |  | IR406 | **ACTG**aagcttGCAGACGCGAGTCCTCAGA |
| MITA | MITApro-luc | MF407  MR408 | **ACTG**ctcgagGTCAATCGTCAACCTAACCAC  TGTCCaagcttTCGATACC |
| IRF3 | IRF3pro-luc | IF1796  IR1827 | **ACTG**ctcgagTGGTAAAGACCAAAGAGAGGGAG  **ACTG**aagcttGTTGCAAACAGACTACACAACACA |
| IRF7 | IRF7pro-luc | IF140  IR141 | **ACTG**ctcgagGTCTTCAGTGCCACATGATC  **ACTG**aagcttTTCAGGTCTTGAAGTAGCTT |
| IFN1 | IFN1pro-luc | IPF1807 | **AT**ctcgagGGTTGGTTTTAAAGTAGGCC |
|  |  | IPR1808 | **ACTG**aagcttGAGAGAATGTTGCAGGGAC |
| IFN2 | IFN2pro-luc | IF293 | **ACTG**ggtaccTCTAGCAATTGTTTCTACACCAA |
|  |  | IR294 | **ACTG**ctcgagGCCACTTGTTTTGATAGTTTGT |
| IFN3 | IFN3pro-luc | IFF568  IFR569 | **GA**agatctTACCAAAACCAGAGCCAAGAG  **CCC**aagcttAAGGAACGGAGAGCGTATGA |
| IFN4 | IFN4pro-luc | IFF570  IFR571 | **GA**agatctTGGGAAATGAACGCAAGGT  **CCC**aagcttTCCACAGGTAGATGAGCCG |
| IFNγ1 | IFNγ1pro-luc | IF381 | **ACTG**ctcgagGCTGGCTGAAATGAAAATC |
|  |  | IR382 | **ACTG**aagcttGGTGAGTGTTGATGATCGG |
| IFNγ2 | IFNγ2pro-luc | IF383 | **ACTG**ctcgagATCTCCTATCAAACGGCTAAAC |
|  |  | IR385 | **ACTG**aagcttTCTTCTGTGCAATCTGACAGC |
| NF-κB1 | NF-κB1pro-luc | NF295 | **ACTG**ctcgagACAAACCAGTCTGTTATGGCC |
|  |  | NR296 | **ACTG**aagcttGCAATTGTGACAATATGCAAGG |
| NF-κB2 | NF-κB2pro-luc | NF272 | **ACTG**ggtaccACTGTCTTTAATGTCCCGTAT |
|  |  | NR273 | **ACTG**ctcgagTCCATATAAGAAAGCAGACTATT |
| RIG-I | RIG-I-HA | RF416 | **ACTG**ggtaccATCGCTGCAAAAATGTACGAG |
|  |  | RR300 | **ACTG**gggcccTTA*AGCGTAGTCTGGGACGTCGTATGGGTA*TTGTGTTTGCCGCCCACA |
|  | RIG-I-Flag | RF416 | **ACTG**ggtaccATCGCTGCAAAAATGTACGAG |
|  |  | RR670 | **ACTG**gggcccTTA*CTTATCGTCGTCATCCTTGTAATC*TTGTGTTTGCCGCCCACA |
| MDA5 | MDA5-HA | MF414 | **ACTG**ggtaccTCAAAAATGAGTAGTGATCAGGA |
|  |  | MR298 | **ACTG**gggcccTTA*AGCGTAGTCTGGGACGTCGTATGGGTA*TAAGTGTTTTTTCTTACAGTACTCTGTT |
|  | MDA5-Flag | MF414 | **ACTG**ggtaccTCAAAAATGAGTAGTGATCAGGA |
|  |  | MR669 | **ACTG**gggcccTTA*CTTATCGTCGTCATCCTTGTAATC*TAAGTGTTTTTTCTTACAGTACTCTGTT |
| RIG-I-CARDs | RIG-I-CARD-HA | RF416 | **ACTG**ggtaccATCGCTGCAAAAATGTACGAG |
|  |  | RR509 | **ACTG**gggcccTTA*AGCGTAGTCTGGGACGTCGTATGGGTA*TCGATCATGCTCCCCTGTG |
|  | RIG-I-CARD-Flag | RF416 | **ACTG**ggtaccATCGCTGCAAAAATGTACGAG |
|  |  | RR783 | **ACTG**gggcccTTA*CTTATCGTCGTCATCCTTGTAATC*TCGATCATGCTCCCCTGTG |
| RIG-I-DExD/H helicase | RIG-I-Helicase-HA | RF510 | **ACTG**ggtaccATGCTAGGTGAGATTAAGCTAAGGGA |
|  |  | RR511 | **ACTG**gggcccTTA*AGCGTAGTCTGGGACGTCGTATGGGTA*ACTGGAGATCAGGAAACACC |
| RIG-I-RD | RIG-I-RD-HA | RF512 | **ACTG**ggtaccATGTCCAGTAGTAAGGAATGCATTGAG |
|  |  | RR300 | **ACTG**gggcccTTA*AGCGTAGTCTGGGACGTCGTATGGGTA*TTGTGTTTGCCGCCCAC |
| MDA5-CARDs | MDA5-CARDs-HA | MF414 | **ACTG**ggtaccTCAAAAATGAGTAGTGATCAGGA |
|  |  | MR513 | **ACTG**gggcccTTA*AGCGTAGTCTGGGACGTCGTATGGGTA*TGCAGACACACTCAGAACGC |
|  | MDA5-CARD-Flag | MF414 | **ACTG**ggtaccTCAAAAATGAGTAGTGATCAGGA |
|  |  | MR782 | **ACTG**gggcccTTA*CTTATCGTCGTCATCCTTGTAATC*TGCAGACACACTCAGAACGC |
| MDA5-DExD/H helicase | MDA5-Helicase-HA | MF514 | **ACTG**ggtaccATGTGCGTTCTGAGTGTGTCTGC |
|  |  | MR515 | **ACTG**gggcccTTA*AGCGTAGTCTGGGACGTCGTATGGGTA*GGCTCGATTCATCTTGCA |
| MDA5-RD | MDA5-RD-HA | MF516 | **ACTG**ggtaccATGCTGATGAATCGAGCCGACTAC |
|  |  | MR517 | **ACTG**gggcccTTA*AGCGTAGTCTGGGACGTCGTATGGGTA*GTTACAGTACTCTGTTCTGTGAAAT |
| LGP2 | LGP2-Flag | LF461 | **ACTG**ggtaccACACAGACCTCGACGCTA |
|  |  | LR107 | **ACTG**gggcccTTA*CTTATCGTCGTCATCCTTGTAATC*GTGACAGTCGTCTAAGTCCA |

Note: The nucleotides in overstriking represent protective bases, and in lowercase indicate the sites of restricted enzymes. Those in italic stand for HA or Flag tag sequence. The nucleotide sequence IDs are as follows: RIG-I promoter JX649222; MDA5 promoter JN986720; IPS-1 promoter KY613777; MITA promoter KY613778; IRF3 promoter KY613779; IRF7 promoter KY613780; IFN1 promoter GU139255; IFN2 promoter KY613781; IFN3 promoter KY613782; IFN4 promoter KY613783; IFNγ1 promoter KY613784; IFNγ2 promoter KY613785; NF-κB1 promoter KY613786; NF-κB2 promoter KY613787; RIG-I ORF GQ478334; MDA5 ORF FJ542045; LGP2 ORF FJ813483.

**
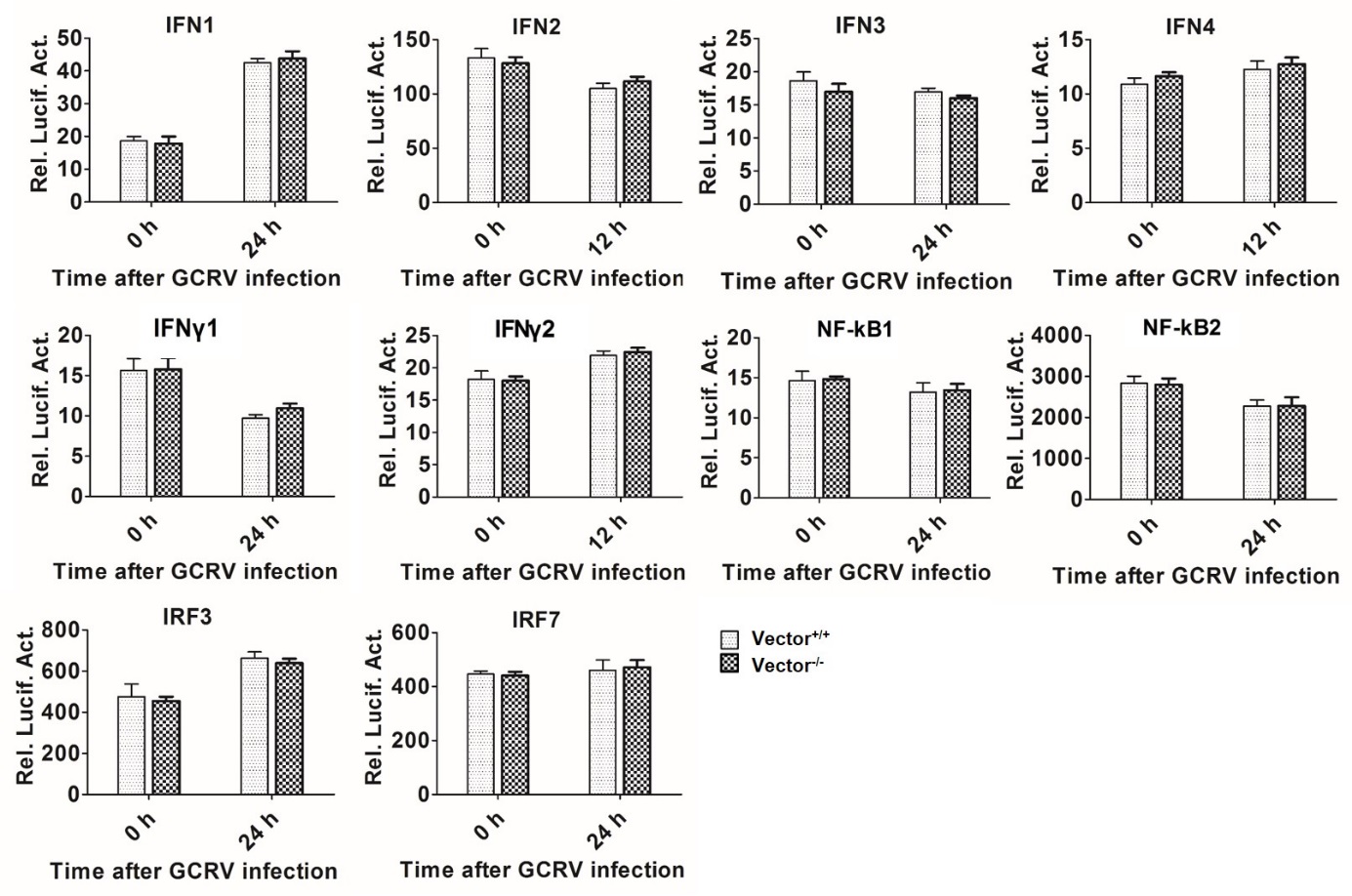
**

**Supplemental Figure 1** Empty vector (pCMV-CMV-EGFP) had no influence on the promoter activations of IFNs, NF-κBs, IRF3 and IRF7. FHM cells were cotransfected with or without 300 ng empty vector (pCMV-CMV-GFP), 30 ng of pRL-TK and 300 ng of IFN1pro-luc, IFN2pro-luc, IFN3pro-luc, IFN4pro-luc, IFNγ1pro-luc, IFNγ2pro-luc, NF-κB1pro-luc, NF-κB2pro-luc, IRF3pro-luc and IRF7pro-luc in 24-well plates. pRL-TK was served as an internal control. At 16 h post-transfection, the cells were infected with GCRV at indicated time points. Then the cells were collected for dual-luciferase report assays. Error bars indicate standard deviation (n = 4).

**
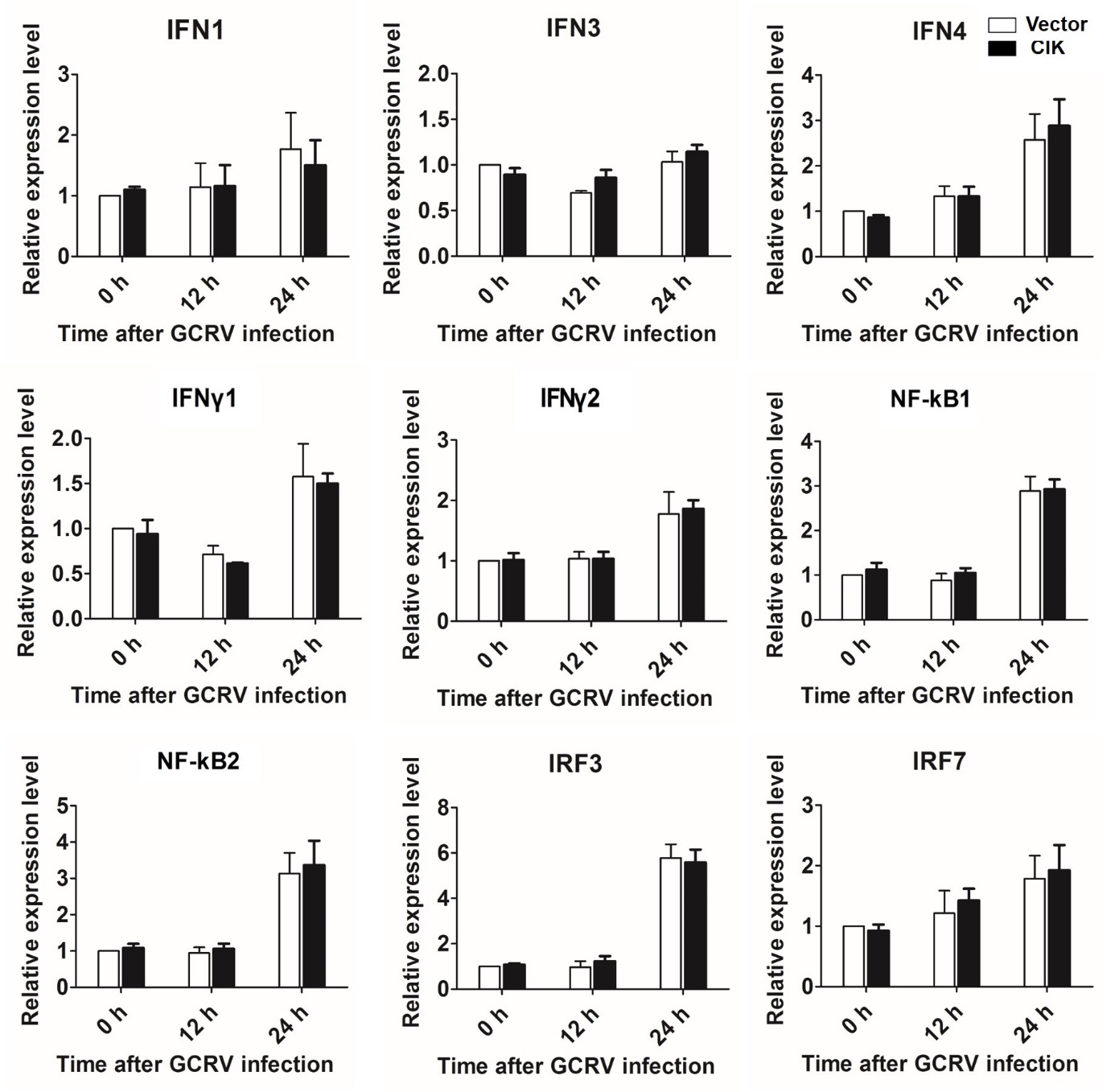
**

**Supplemental Figure 2** Comparison of the expression patterns of IFNs, NF-κBs, IRF3 and IRF7 between CIK and empty vector transfected cells. Normal CIK cells and empty vector transfected CIK cells were infected with GCRV for 12 h and 24 h in 12 well-plates. Then the cells were harvested for qRT-PCR to quantify the relative expression levels of IFN1, IFN3, IFN4, IFNγ1, IFNγ2, NF-κB1, NF-κB2, IRF3 and IRF7, respectively. Error bars indicate standard deviation (n = 4).


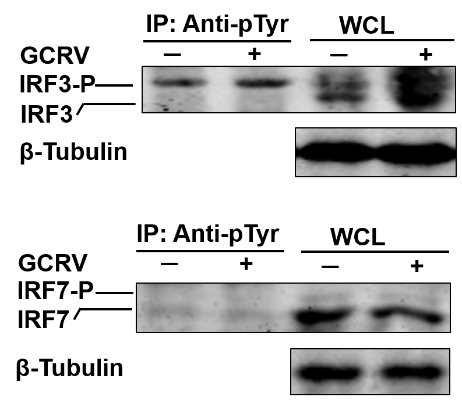


**Supplemental Figure 3** Tyr phosphorylation is not a target for virus-induced phosphorylation of IRF3 and IRF7. CIK cells were plated in 10-cm^2^ dishes and infected with or without GCRV. At 24 h post GCRV infection, the cells were harvested for IP with anti-pTyr Ab. IB was performed with anti-IRF3 and anti-IRF7 antiserum and β-Tubulin Abs.


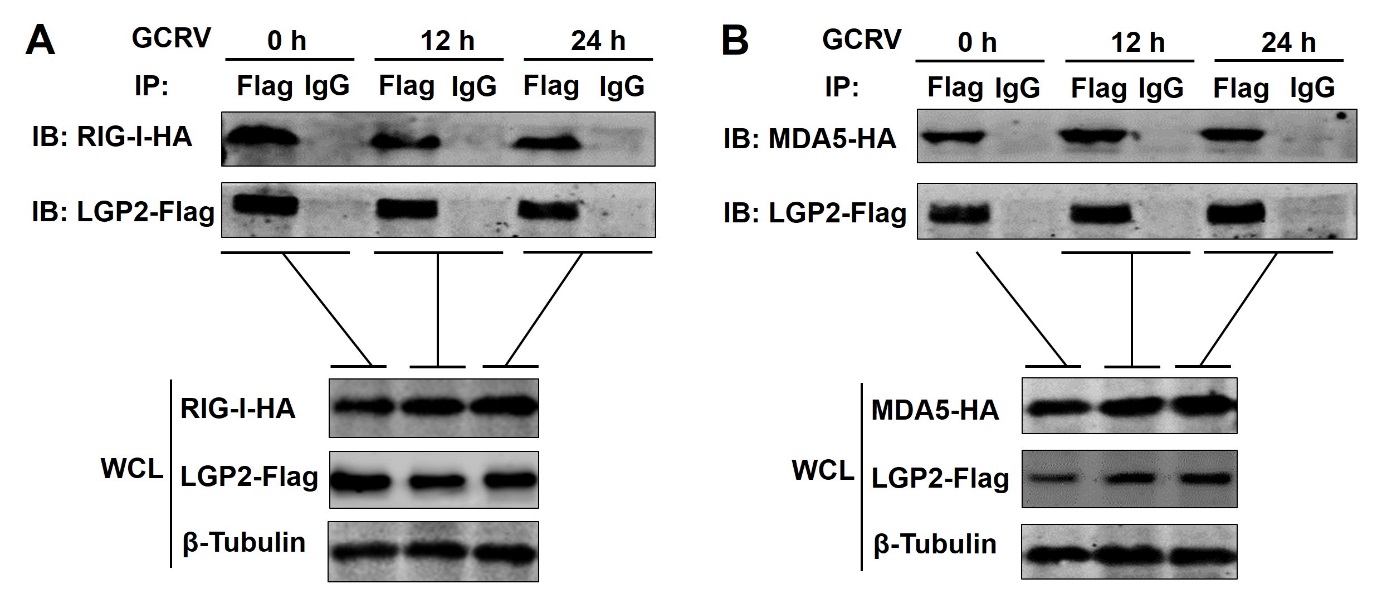


**Supplemental Figure 4** Verification of the interaction between LGP2 and RIG-I or MDA5. FHM cells were plated in 10-cm^2^ dishes for 24 h. Then the cells were cotransfected with 4 μg LGP2-Flag and 4 μg RIG-I-HA (**A**), or 4 μg LGP2-Flag and 4 μg MDA5-HA (**B**) for 16 h, and then uninfected or infected with GCRV for 12 h and 24 h. Co-IP and IB were performed with the indicated Abs. WCL was subjected to IB with anti-Flag, anti-HA and anti-β-Tubulin Abs, respectively.


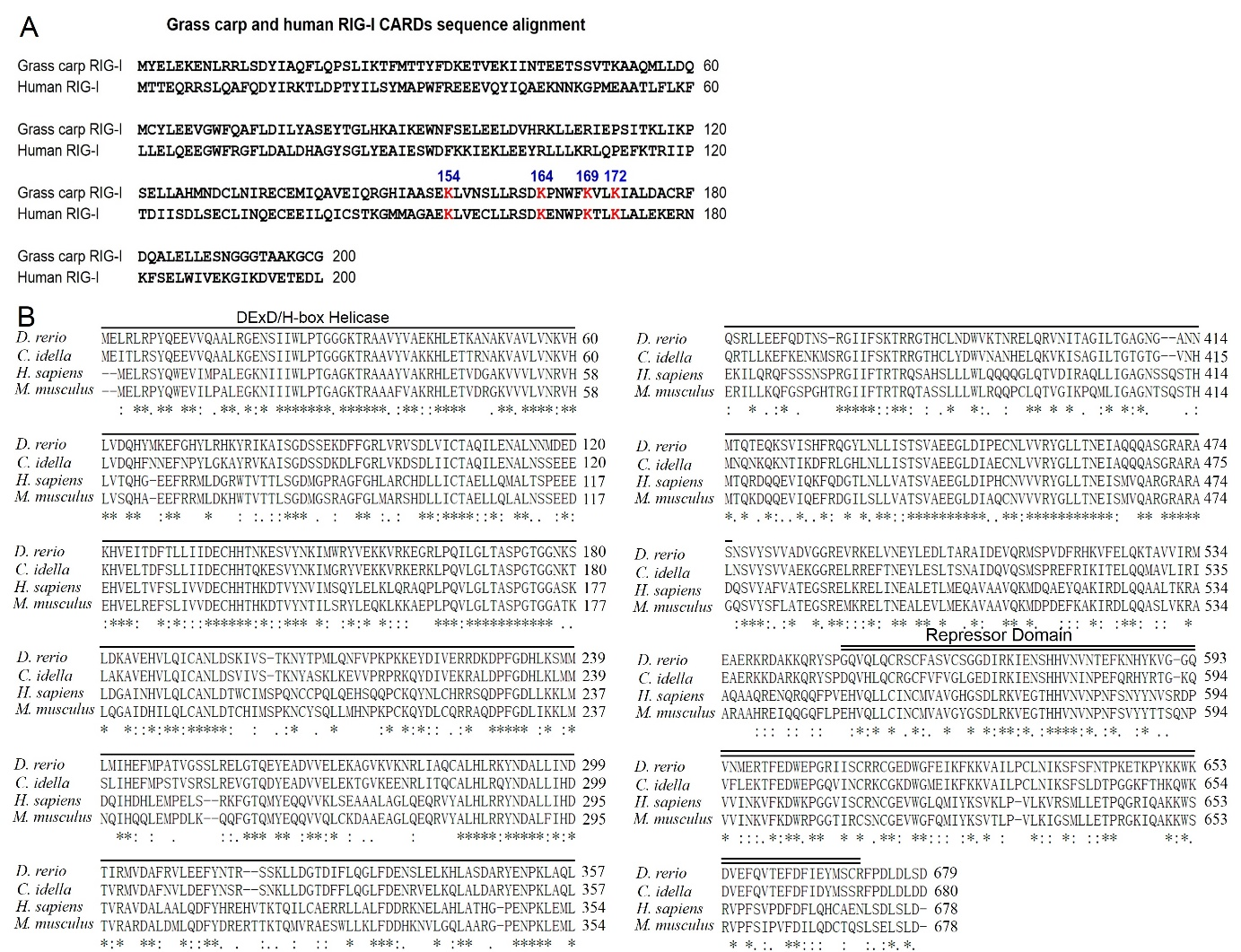


**Supplemental Figure 5** (**A**) Sequence alignment of grass carp and human RIG-I CARDs (GenBank: AAD19826). Alignment was performed using ClustalW (<http://www.genome.jp/tools/clustalw/>). The lysine residues in red indicate the conserved sites of ubiquitination between grass carp and human RIG-I CARDs, and the corresponding positions are highlighted with numbers. (**B**) Amino acid alignment of LGP2 homologs from different species. Alignment was performed using ClustalW (<http://www.genome.jp/tools/clustalw/>). The protein IDs are as follows: *Homo sapiens* (*H. sapiens*) LGP2 NP_077024; *Mus musculus* (*M. musculus*) LGP2 AAH29209; *Danio rerio* (*D. rerio*) LGP2 AKA09351; *Ctenopharyngodon idella* (*C. idella*) LGP2 AFQ93565.


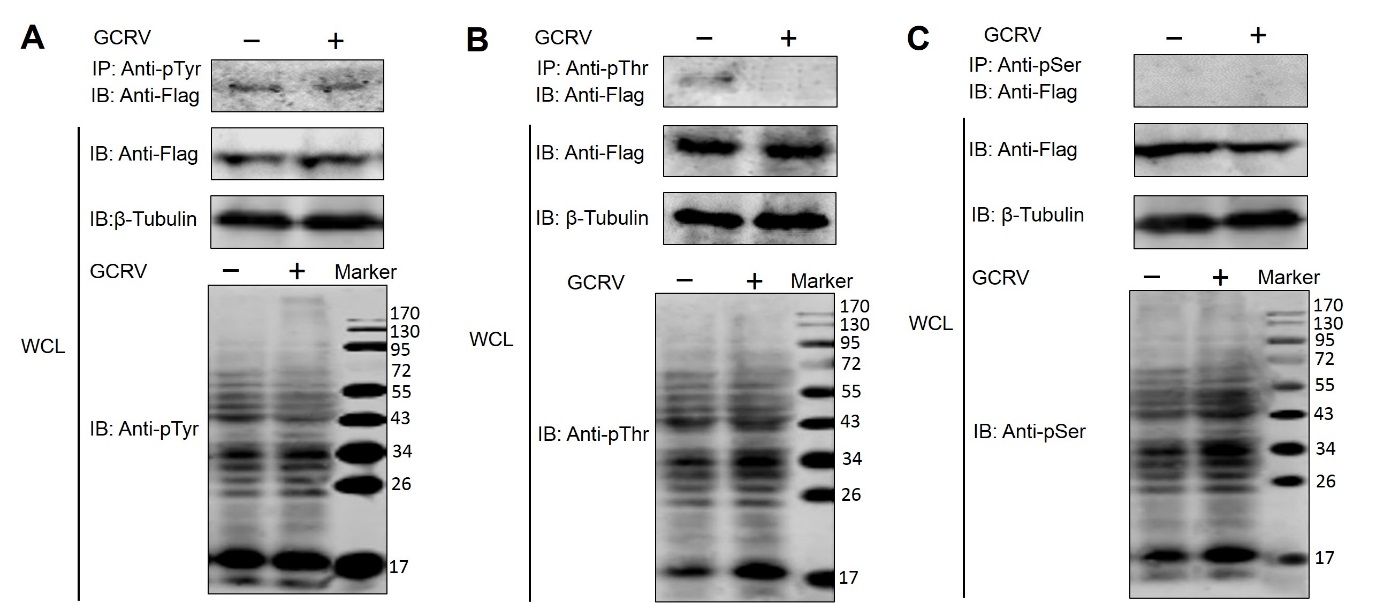


**Supplemental Figure 6** LGP2 involves in phosphorylation modification. LGP2-Flag stable transfected CIK cells were seeded in 10-cm^2^ dishes for 24 h. Then the cells were infected with or without GCRV for 24 h, and the cells were lysed for IP with anti-pTyr (**A**), anti-pThr (**B**) and anti-pSer (**C**) Abs, respectively. The IP samples and WCL were subjected to IB with the indicated Abs.
